# Recent Contributions of Organic Synthesis to Forensic Science

**DOI:** 10.1002/cplu.202500724

**Published:** 2026-02-19

**Authors:** Gustavo dos Santos Martins, Mariana dos Santos Dupim, Rodrigo Garcia Agostinho, Fernanda Gadini Finelli

**Affiliations:** ^1^ Instituto de Pesquisas de Produtos Naturais Walter Mors Universidade Federal do Rio de Janeiro Rio de Janeiro Brazil

**Keywords:** amphetamine, cannabinoids, chemical profiling, fentanyl, new psychoactive substances

## Abstract

Forensic science is a multidisciplinary field that plays a vital role in society by supporting criminal investigations and providing scientific evidence for judicial proceedings. Within this context, organic chemistry contributes fundamentally by enabling the structural elucidation of seized drugs and their intermediates, the synthesis of analytical reference standards for doping control, allied with a deeper understanding of drug metabolization, and the development of probes for detecting fingerprints and biological fluids. This review examines recent advances at the interface between organic synthesis and forensic science. The discussion is organized into three main application areas: drug identification, toxicology, and fingerprint analysis, highlighting how synthetic methodologies have been employed to address key challenges in each topic. Furthermore, this work aims to shed light on the broad range of opportunities for the organic synthesis community to contribute to the advancement of forensic science.

## Graphical abstract

Forensic science is a multidisciplinary field that plays a vital role in providing scientific evidence for criminal investigations. This review highlights advances and opportunities at the interface between organic synthesis and forensic science, with applications in drug identification, forensic toxicology, and latent fingerprint detection and visualization.

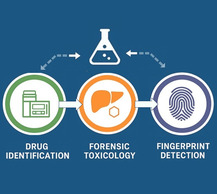

## 1 Introduction

Forensic science is a multidisciplinary field grounded in scientific methods and principles that support decision‐making within the justice system. It is routine practice to involve the observation, documentation, collection, analysis, and interpretation of criminal or civil evidence using precise, reliable, and rapid procedures. As criminal scenarios become increasingly complex, driven by the continuous emergence of new psychoactive substances and the growing sophistication of illicit processes, the need for tools capable of accurately determining the composition, origin, and chemical transformations of substances associated with criminal activities becomes more pressing. In this context, advanced approaches are crucial for expanding investigative capabilities and meeting increasingly demanding analytical challenges [1, 2].

Organic synthesis, with its ability to construct, modify, and predict molecular architectures, holds a strategic position in this field. Drug identification in seized materials, one of the most frequently requested forensic analyses, is typically performed by comparing chromatographic and mass spectrometric data with reliable standards [3, 4]. In such cases, the production of reference standards provides precision and traceability to analytical procedures, thereby preventing the invalidation of evidence during legal proceedings due to methodological weaknesses [5].

The synthesis of reference standards is also crucial for identifying drug metabolites present in biological samples. This step plays a central role in postmortem investigations to determine the involvement of substances in deaths; in studies of performance impairment and drug‐facilitated crimes to assess the impact of substances on human performance; in doping control, for detecting performance‐enhancing drugs; and in workplace drug testing programs designed to identify substance use by employees [2].

Another relevant area of intersection between organic synthesis and forensic science is the chemical profiling of seized materials. These chemical signatures provide useful information for forensic chemists and law enforcement agencies. The analysis of detected and characterized impurities allows organic chemists to gain insights into the synthetic methods and precursors used, contributing to the identification of clandestine laboratories, trafficking routes, and the geographic origins of seized drugs, as well as supporting the classification of controlled substances by authorities [3, 6].

Finally, the identification and collection of evidence at crime scenes are crucial steps for reconstructing events and identifying suspects. Fingerprints can be revealed quickly and inexpensively, providing individual identification comparable to that obtained through DNA analysis. However, most fingerprints are invisible to the naked eye and require development processes to make their patterns visible. In this scenario, the growing exploration of organic synthesis is for the design of fluorescent probes capable of detecting and revealing latent fingerprints through selective interactions with their chemical constituents [7, 8].

This review examines recent advances at the interface between organic synthesis and forensic science over the last 5 years. The discussion is organized into three main application areas: drug identification, forensic toxicology, and crime scene, represented by latent fingerprint development. It outlines the synthetic strategy behind each example, highlighting the key transformations involved and the improvements achieved for each application. We hope to draw the attention of the scientific community, presenting a big‐picture view to pave the way for more efficient and accurate forensic methodologies. By consolidating these developments, this review highlights emerging opportunities for innovation at the synthesis‐forensics interface.

## 2 Drug Identification

Drug identification is an essential step in forensic investigations and in the control of illicit substances, allowing not only the detection and classification of known compounds but also the characterization of new synthetic drugs and their precursors. This process involves the integration of analytical techniques and organic synthesis expertise, which are essential for elucidating production pathways, and chemical modifications involved in the creation of analogs and derivatives of controlled substances, assisting in the prediction and identification of emerging compounds. In this context, the Drug Identification section of this review is subdivided into three main topics: controlled substances, already known and regulated by global regulatory frameworks, whose identification supports legal control, verification of authenticity, and monitoring of drug trafficking patterns; new psychoactive substances (NPS), emerging compounds that mimic the effects of traditional drugs, whose detection enables early recognition of new threats and adaptation of analytical methodologies; and chemical profiling, which enables the identification of chemical constituents to trace origins, synthetic routes, and adulterants in illicit formulations, thereby linking evidence from crime scenes to specific sources, production methods, or other related samples.

### 2.1 Controlled Substances

**SCHEME 1:**
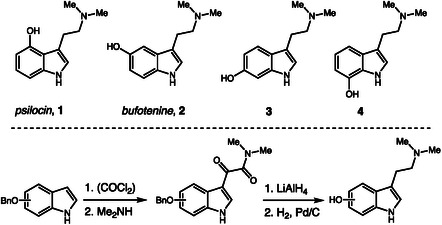
Synthesis of psilocin and bufotenine isomers.

In 2020, Mishraki‐Berkowitz and coworkers addressed a classic challenge in forensic chemistry by accurately distinguishing structural isomers of psychoactive controlled substances [9]. Psilocin (**1**) and bufotenine (**2**) are naturally occurring hallucinogen compounds, while their synthetic isomers, 6‐hydroxy‐*N*,*N*‐dimethyltryptamine (**3**) and 7‐hydroxy‐*N*,*N*‐dimethyltryptamine (**4**), are not controlled but can be easily misidentified due to their structural similarity (Scheme 1). To prevent such errors, the authors synthesized these isomers starting from the hydroxyindole derivatives, following the classical condensation and reduction steps originally described by Hofmann in his pioneering work on psilocybin [10]. They then compared the profiles of all four compounds using routine laboratory techniques including TLC, FTIR, and GC–MS. The results demonstrated that combining TLC and GC–MS enables clear differentiation of the isomers, with TLC providing rapid visual identification, GC–MS confirming compound identity, and FTIR proving effective when purified samples are used.

**SCHEME 2:**
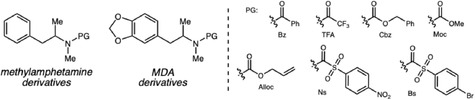
*N*‐Protected methylamphetamine and MDMA derivatives.

Following this classical analytical challenge involving naturally occurring tryptamines, Xu and coworkers expanded the discussion to synthetic amphetamine‐type stimulants (ATS), focusing on strategies used to chemically mask controlled substances through structural modification [11]. They synthesized a series of methylamphetamine and MDMA containing benzoyl (Bz), trifluoroacetyl (TFA), carboxybenzyl (Cbz), allyloxycarbonyl (Alloc), methoxycarbonyl (Moc), nitrobenzenesulfonyl (Ns), and bromobenzenesulfonyl (Bs) protecting groups (Scheme 2). These compounds were analyzed by GC–MS, HRMS, NMR, FTIR, and color tests to create a robust analytical dataset. The study revealed that these modified compounds maintain high yields and purity and can easily revert to the parent drug under mild deprotection conditions, confirming their potential as concealed precursors for illicit ATS synthesis.

**SCHEME 3:**
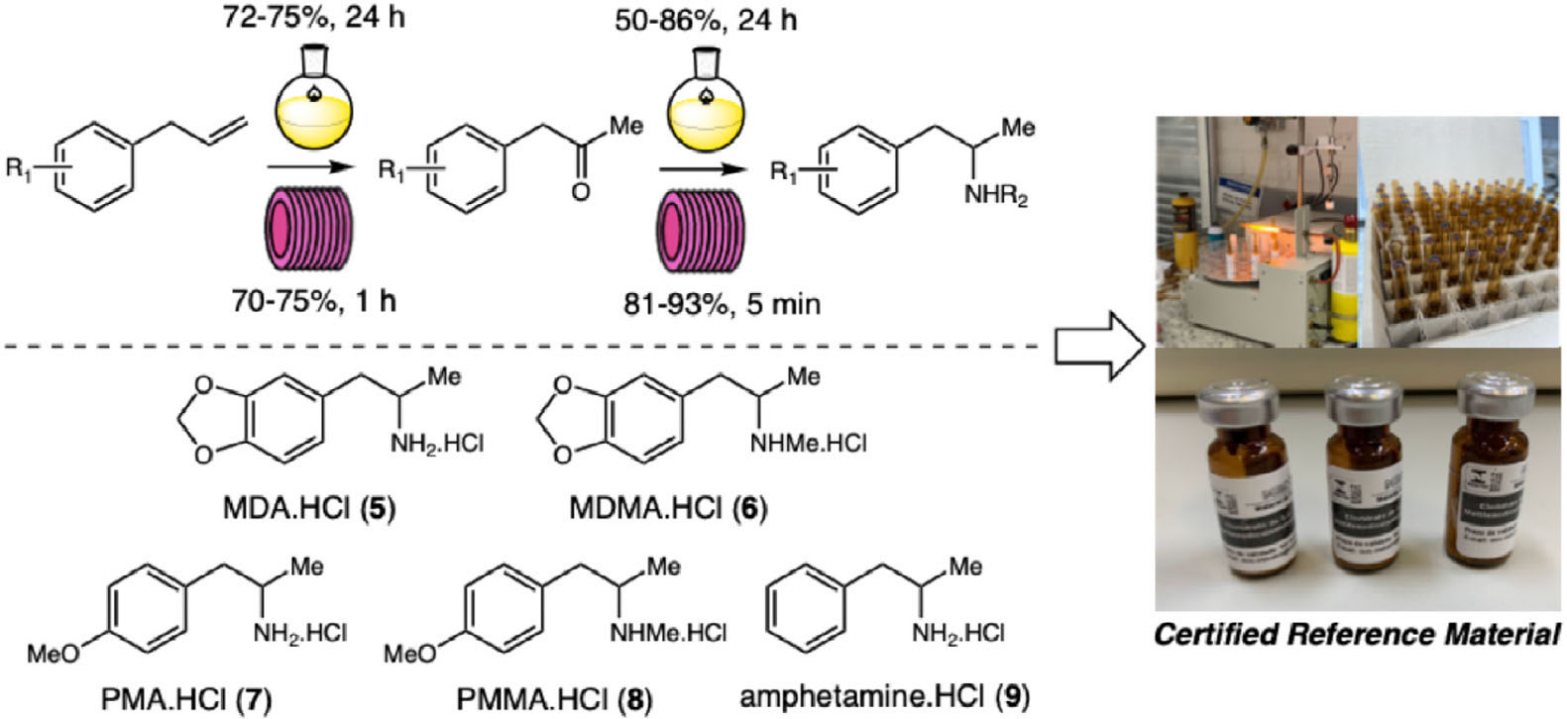
Developing amphetamine Certified Reference Materials.

Subsequently, our group developed a concise and efficient synthetic route for the amphetamine derivatives MDA (**5**), MDMA (**6**), PMA (**7**), and PMMA (**8**), via Wacker–Tsuji oxidation followed by reductive amination (Scheme 3). Comparative studies under batch and continuous‐flow conditions revealed that flow chemistry dramatically enhanced reaction efficiency, significantly reducing synthesis time and improving overall yields [12]. Beyond these synthetic advances, the study also established a comprehensive certification protocol for amphetamine Certified Reference Materials (CRMs), which are homogeneous and stable substances with well‐defined purity that play a crucial role in ensuring analytical quality, method validation, and metrological traceability. The studies involved rigorous assessments of identity, homogeneity, stability, and purity using NMR, LC‐MS/MS, and qNMR techniques. The resulting MDA.HCl CRM achieved a certified purity of 99.1 ± 1.4 g/100 g and was transferred to the Brazilian Federal Police, used for analytical calibration and quality assurance in forensic laboratories.

Building upon these studies, in 2025, an amphetamine.HCl (**9**) CRM was synthesized on a 3 g scale and fully certified, marking a further step toward establishing Brazilian capacity for CRM production and reinforcing the forensic metrology infrastructure of the country [5].

**SCHEME 4:**
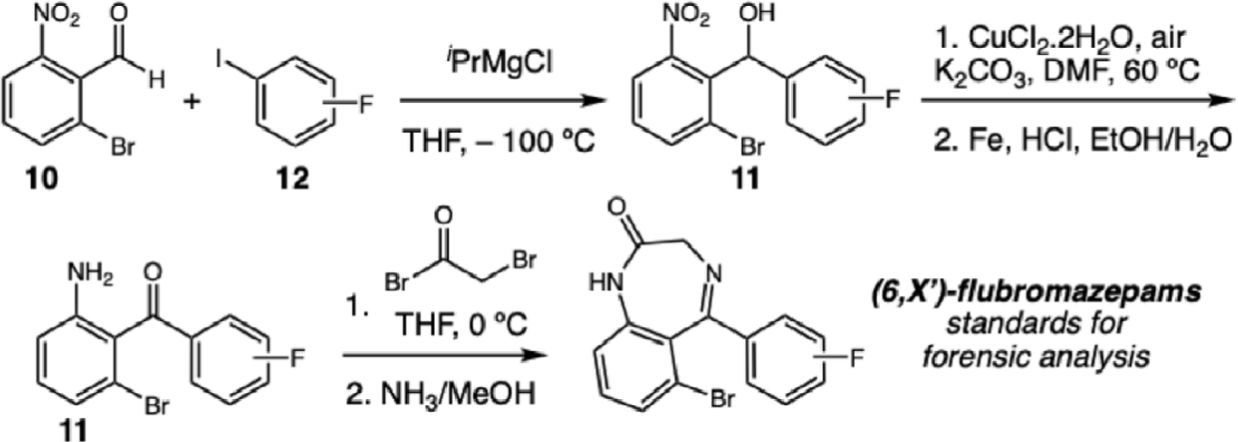
Synthesis of (6,X’)‐flubromazepam derivatives.

In 2022, France and Parker developed a versatile synthetic methodology for the preparation of psychotropic benzodiazepine derivatives, aiming to overcome the shortage of analytical reference standards that hinders their identification in forensic contexts (Scheme 4) [13]. Their approach utilizes common intermediates to access structurally diverse compounds, resulting in a faster, more economical, and standardized process compared to conventional compound‐specific synthetic routes. The strategy involves a Grignard addition to aldehyde **10**, followed by oxidation of the resulting hydroxyl group and reduction of the nitro group to yield intermediate **11**. Subsequent acylation and condensation with ammonia afford the (6,X’)‐flubromazepam derivatives. Structural diversification is achieved by modifying the functional group on the starting aryliodide **12**, enabling the introduction of substituents at key positions within the benzodiazepine scaffold. The target compounds were obtained in good yields and fully characterized by NMR, HRMS, and HPLC analyses. The synthesized derivatives served as analytical reference standards for the identification of compounds detected in seized materials, allowing confirmation of unknown structures and validation of forensic screening methodologies.

Extending the concept of reference material production beyond synthetic analogs, dos Santos and coworkers reported the isolation and characterization of cocaine, along with the synthesis of its main metabolite, benzoylecgonine, the methyl ester hydrolysis product, to develop a forensic reference standard directly from seized drug samples [14]. Cocaine was purified from confiscated material using column chromatography, achieving a 50% purification yield and 98.4% purity. From this purified compound, benzoylecgonine is obtained by hydrolyzing cocaine under reflux in water/dioxane, with 91% yield. Both substances were thoroughly characterized using GC–MS, HPLC‐DAD, and NMR techniques, confirming their structural integrity and analytical reliability. Following homogeneity and purity studies, benzoylecgonine exhibited 96.4% purity, validating its suitability as a reference standard for forensic analytical applications.

### 2.2 New Psychoactive Substances

**SCHEME 5:**
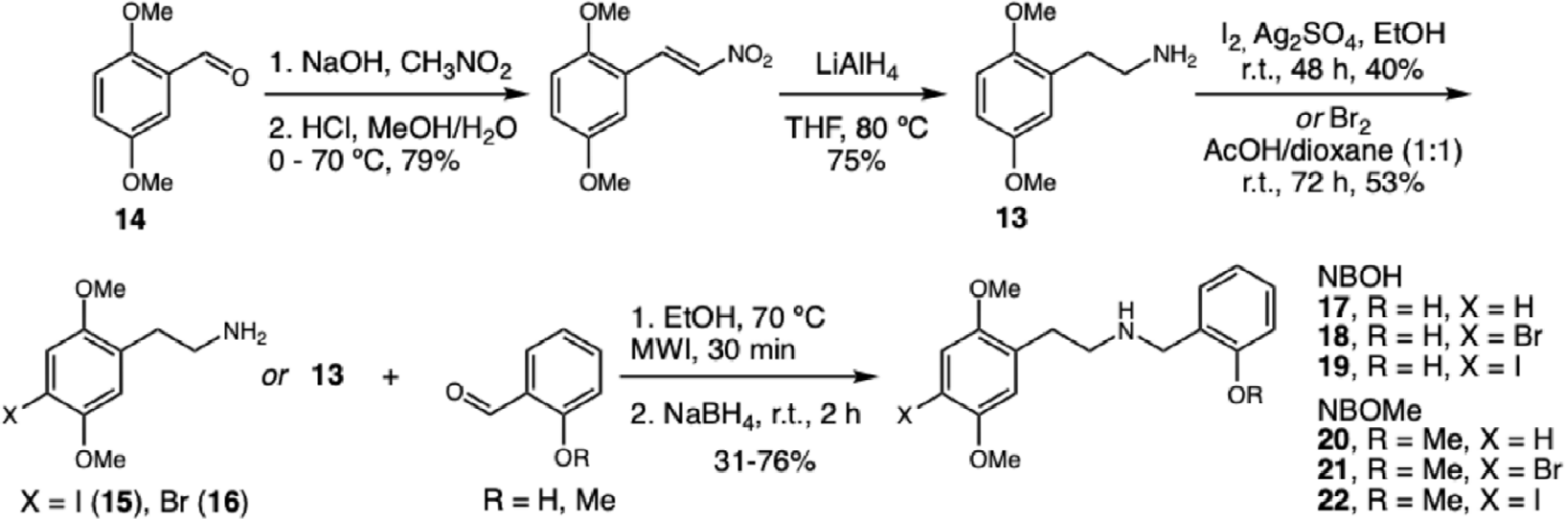
Synthesis of NBOHs and NBOMes.

Among the new psychoactive substances (NPS) reported worldwide, 136 were identified as phenethylamines, including the potent hallucinogens NBOMes and NBOHs. In 2021, de Fátima and coworkers described the synthesis of a series *N*‐benzylphenethylamine derivatives, emphasizing their importance as reference standards for pharmacological studies and forensic analyses (Scheme 5) [15]. The synthetic strategy was based on the preparation of halogenated derivatives from amine **13** as key intermediates, obtained from 2,5‐dimethoxybenzaldehyde (**14**) via the Henry reaction, followed by dehydration and reduction with LiAlH_4_. The halogenation step was conducted using iodine or bromine under different conditions, and the resulting intermediates **15, 16** were subsequently converted into the desired products through Schiff base formation and reductive amination with NaBH_4_ in a single‐pot process under microwave irradiation. This accessible synthetic route enabled the preparation of six compounds with overall yields ranging from 7% to 38%, overcoming previous limitations related to the limited availability and high cost of analytical standards for these substances. Furthermore, the gram‐scale synthesis of NBOH **17–19** and NBOMe **20–22** demonstrated the robustness and reproducibility of the method, confirming its potential for large‐scale application.

**SCHEME 6:**
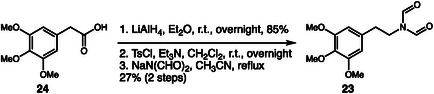
Synthesis of a mescaline analog.

In 2023, Gallagher and coworkers reported the identification, characterization, and synthesis of *N*,*N*‐diformylmescaline (**23**), a NPS analog of mescaline detected for the first time in drug seizures in Queensland, Australia [16]. The investigation began with the analysis of seized samples, which contained a white powder initially suspected to be cocaine. Using GC–MS, NMR, and FTIR, the researchers determined that the substance was in fact a diformylated form of mescaline. To confirm this hypothesis, they developed a three‐step synthetic route, starting from 3,4,5‐trimethoxyphenylacetic acid (**24**) (Scheme 6). The sequence involved reduction of the acid to a primary alcohol using LiAlH_4_ in 85% yield, followed by tosylation of the alcohol to generate a reactive intermediate, and finally a nucleophilic substitution with sodium diformamide in refluxing acetonitrile, yielding **23** with 27% after 2 steps. The results showed that **23** is chemically unstable, readily degrading under acidic or basic conditions to form *N*‐formylmescaline and, subsequently, mescaline. This behavior suggests that the compound may act as a prodrug, releasing mescaline after ingestion, a property that could facilitate its use to circumvent drug control legislation by masking the presence of a controlled substance. Furthermore, the study identified several impurities and byproducts formed during synthesis, providing valuable clues about the potential production route used in clandestine laboratories.

**SCHEME 7:**
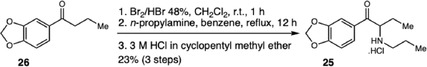
Synthesis of bk‐PBDB.

In a similar effort to strengthen forensic identification, Sutcliffe and coworkers described the synthesis of putylone (bk‐PBDB, **25**), a synthetic derivative of the cathinone family that mimics the psychostimulant effects of MDMA (**6**), whose structure is shown in Scheme 3. This compound was identified for the first time in the United Kingdom in tablets seized and sold as ecstasy [17]. To confirm the structure of this NPS, the authors synthesized a pure reference standard and directly compared the seized samples using ^1^H and ^13^C NMR, FTIR‐ATR, and GC‐EI‐MS analyses. The synthetic route employed involved bromination of the 3,4‐methylenedioxypropiophenone (**26**) precursor, followed by amination with propylamine and subsequent conversion to the hydrochloride salt, yielding a stable and high‐purity product (Scheme 7). The analyses showed strong concordance between the seized material and the synthetic standard and revealed that the seized tablets contained a mixture of bk‐PBDB (130–135 mg per tablet) and caffeine (40–43 mg per tablet). Furthermore, a rapid and sensitive GC‐EI‐MS method was developed and validated for the detection and quantification of bk‐PBDB (**25**), with limits of detection and quantification of 0.09 and 0.26 µg/mL, respectively.

**SCHEME 8:**
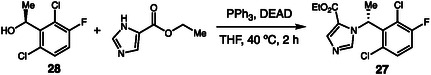
Synthesis of an analog of etomidate.

As the diversity of NPS continues to expand beyond classic stimulant structures, Gao and coworkers reported a novel halogenated analog of the anesthetic etomidate **27**, illustrating the growing chemical complexity of psychoactive agents detected in alternative formulations such as e‐liquid samples seized in China (Scheme 8) [18]. Unlike previously reported derivatives, this compound exhibits uncommon substitutions on the benzene ring, representing a new structural class among etomidate analogs. Structural elucidation was achieved through the synthesis of a reference standard from a Mitsunobu reaction with (*S*)‐1‐(2,6‐dichloro‐3‐fluorophenyl)ethanol (**28**), followed by characterization using GC–MS, UHPLC‐QTRAP‐MS/MS, NMR (^1^H, ^13^C, ^19^F, COSY, HSQC, HMBC, NOE), and FTIR analyses. The analytical data showed perfect agreement with the seized material, confirming the proposed structure and distinguishing the compound from possible regioisomeric analogs. In silico evaluations further indicated that this NPS possesses high lipophilicity, blood–brain barrier permeability, and a significant risk of neurotoxicity, similar to etomidate, along with potential nephrotoxic and respiratory effects.

**SCHEME 9:**
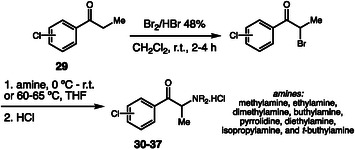
Synthesis of chloro‐cathinones.

In 2025, Pacheco, Gaspar, and coworkers reported the synthesis of a series of chloro‐cathinone derivatives (Scheme 9) [19]. The synthetic route began with the bromination of propiophenones **29**, followed by nucleophilic substitution and to give the target cathinone as free base, followed by acid precipitation to obtain its hydrochloride salt **30–37**. These NPS were characterized using NMR, GC–MS, and HRMS. Furthermore, the study highlighted that the synthesized compounds exhibited significant toxicity in SH‐SY5Y neuronal cells and inhibitory activity toward the enzyme acetylcholinesterase, indicating potential interference with cholinergic neurotransmission.

**SCHEME 10:**
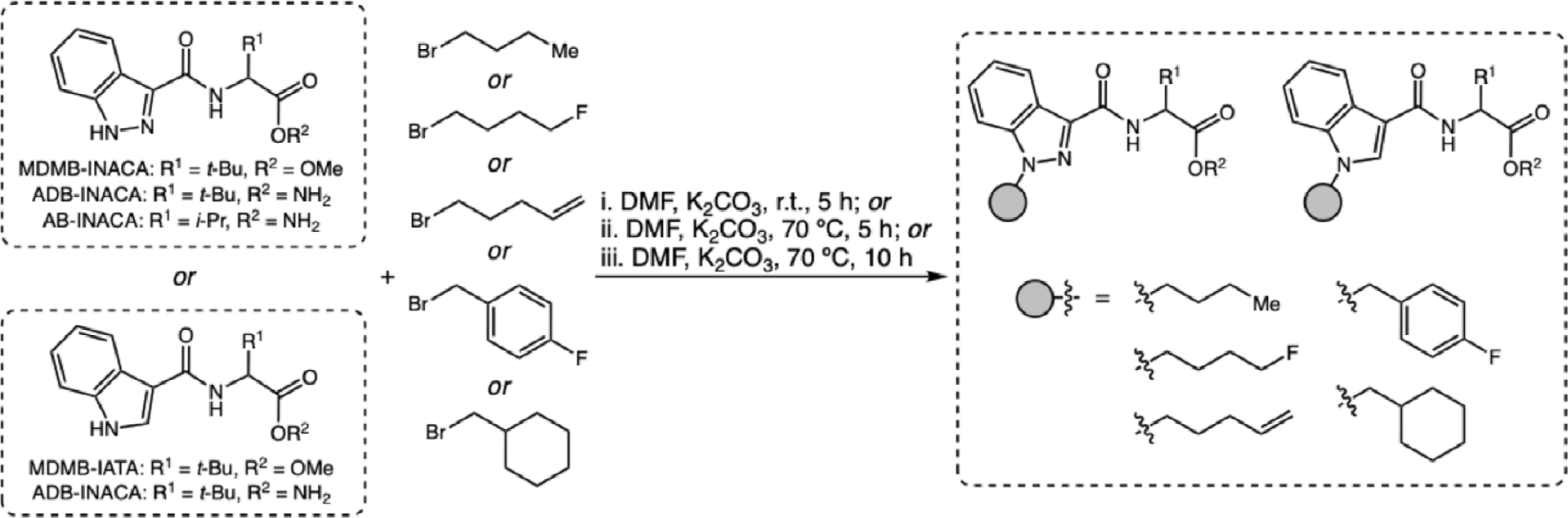
Tail‐less precursors in SCRA production.

Monti and coworkers presented a one‐step synthesis to produce synthetic cannabinoids (SCRAs) from tail‐less precursors, which are uncontrolled compounds that can be converted into banned SCRAs through a simple alkylation reaction (Scheme 10) [20]. This route was identified in a clandestine laboratory in Switzerland and experimentally reproduced, in which the precursors were reacted with the corresponding bromides in the presence of K_2_CO_3_ in DMF, either at room temperature for 5 h or at 70°C for 5–10 h. The procedure yielded products with purity levels ranging from 39% to 93% and varying physical characteristics, such as oils, waxes, or powders. Forensic analyses showed that mixtures of precursors and final SCRAs were found in samples seized in the US and Scotland between 2023 and 2024, demonstrating the international spread of this technique. Biological assays demonstrated that the precursors exhibit low activity at the CB1 receptor, whereas the final products regain the high potency typical of synthetic cannabinoids, which is responsible for severe intoxications. The study concludes that this new synthetic route, which emerged after the 2021 Chinese ban, explains the reappearance of previously banned SCRAs and warns of the need for continuous monitoring, as the increasing use of this method may lead to greater domestic and global production of SCRAs.

### 2.3 Chemical Profiling

**SCHEME 11:**
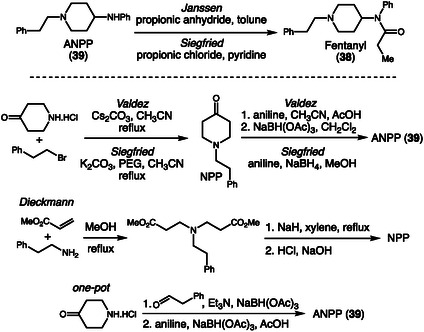
Fentanyl and precursor synthesis.

In 2021, Ovenden and coworkers investigated the synthetic routes employed in the illicit production of fentanyl (**38**) and its precursor 4‐anilino‐*N*‐phenethylpiperidine (ANPP, **39**), focusing on the relationship between synthetic methodology and impurity profiles [21]. Fentanyl, a highly potent synthetic opioid, can be obtained through different synthetic pathways, the most common being the Janssen and Siegfried routes, which differ mainly in the acylating agent used (Scheme 11). The two ANPP acylation strategies were studied and their respective impurity profiles compared. In addition, to ensure the specificity of the identified compounds, the authors also synthesized ANPP (**39**) following the Siegfried, Valdez, Dieckmann, and one‐pot method. As a result, 21 impurities specific to the Siegfried route and 22 to the Janssen route were detected, along with distinct markers for each of the alternative ANPP synthesis routes. Sample characterization was performed by LC‐HRMS combined with multivariate statistical analysis. The reproducibility of the synthetic procedures demonstrated that even small variations in reaction conditions, such as the type of acylating agent or the purification sequence, produce consistent and characteristic impurity profiles for each route.

**SCHEME 12:**
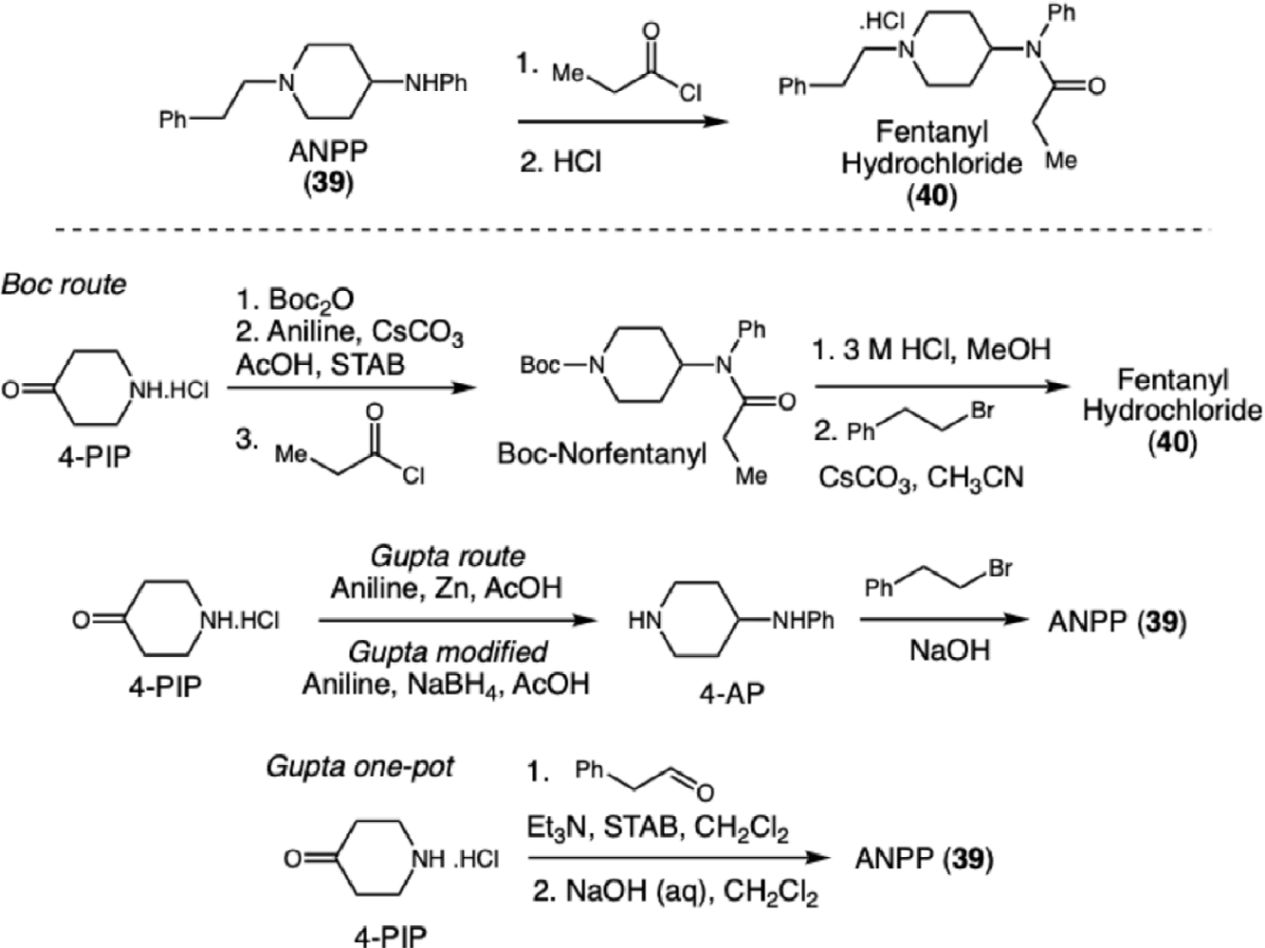
Trends developed in the fentanyl synthesis routes.

Building upon the identification of route‐specific impurities in fentanyl synthesis, Toske and coworkers expanded upon this approach by investigating six distinct synthetic routes for fentanyl hydrochloride (**40**) production, aiming to compare laboratory‐generated organic impurity profiles with those observed in seized samples (Scheme 12) [22]. Using advanced analytical techniques such as GC–MS, NMR, and DART‐MS, the researchers successfully isolated and characterized specific impurities that act as route markers, compounds that reveal the synthetic pathway employed in clandestine laboratories. This strategy enabled the identification of a transition in illicit production practices, highlighting the growing adoption of the Gupta route and its recent modifications, evidenced by the emergence of novel impurities such as phenethyl‐4‐ANPP and ethyl‐4‐ANPP.

**SCHEME 13:**
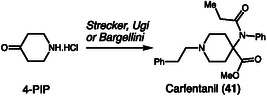
Carfentanil synthesis methods based on chemical impurity profile.

This strategy of route attribution through impurity profiling was further refined by Ostin and coworkers, who demonstrated its robustness by classifying carfentanil (**41**) synthesis methods based on characteristic impurity patterns, synthesizing this ultra‐potent opioid via three distinct pathways, Strecker, Ugi, and Bargellini (Scheme 13) [23]. Notably, the specific reaction conditions for these synthetic routes were not described in the original article. Using GC–MS and UHPLC‐HRMS, the authors analyzed 54 samples and applied multivariate statistical methods, such as orthogonal partial least squares discriminant analysis (OPLS‐DA), to differentiate synthesis routes according to their characteristic impurity profiles. The resulting classification model showed clear separation between the routes and correctly predicted the synthesis method for all samples in an independent validation set, demonstrating both analytical robustness and reproducibility. Beyond laboratory conditions, the study extended its forensic relevance by evaluating whether impurity profiles could still be detected from carfentanil residues on various indoor surfaces, simulating realistic post‐incident scenarios. Samples collected from cotton fabric and wooden surfaces up to 1 week after dispersion were successfully classified by synthesis route, proving the applicability of the method to trace‐level environmental samples.

**SCHEME 14:**
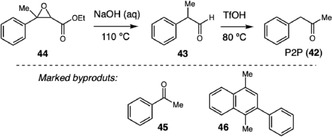
P2P synthesis and forensic markers.

In 2022, Biddle and coworkers presented a comprehensive analysis of the synthetic routes leading to the formation of 1‐phenyl‐2‐propanone (**42**, P2P), a key precursor of methamphetamine used in various synthetic pathways, from both controlled and uncontrolled substances (Scheme 14) [24]. The study demonstrated that 2‐phenylpropionaldehyde (**43**) and ethyl methylphenylglycidate (**44**) can be converted into P2P through acid‐catalyzed rearrangements, particularly under extremely acidic conditions. Systematic experiments varying temperature, reaction time, and acid strength, combined with density functional theory calculations, revealed that the conversion proceeds via a rearrangement mechanism involving double protonation of the aldehyde, which explains the requirement for superacids to achieve efficient transformation. In addition to confirming the potential of these synthetic routes for illicit methamphetamine production, the study identified consistent side products, such as acetophenone (**45**) and 1,4‐dimethyl‐2‐phenylnaphthalene (**46**), which stand out as valuable forensic markers for the chemical profiling of seized samples.

**SCHEME 15:**
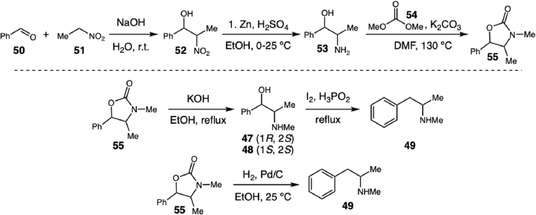
Ephedrine, pseudoephedrine, and methamphetamine synthesis.

Subsequently, Miller and coworkers studied an alternative synthetic pathway to ephedrine (**47**), pseudoephedrine (**48**), and methamphetamine (**49**) from a Henry condensation of benzaldehyde (**50**) and nitroethane (**51**) to give **52**, which is then reduced to **53** and converted by cyclization‐methylation with dimethyl carbonate (**54**) into the oxazolidinone **55**, an intermediate that yields methamphetamine by catalytic hydrogenolysis or ephedrine and pseudoephedrine by basic hydrolysis (Scheme 15) [25]. The study systematically explored different reduction conditions, demonstrating how subtle changes in temperature, acidity, and solvent composition drastically affect the impurity formation. Importantly, the authors emphasize that this route employs largely noncontrolled reagents, increasing its attractiveness to illicit operators; they also note that some impurities lack route specificity and that *N*‐hydroxymethamphetamine can arise from postsynthetic *N*‐oxidation (e.g., decontamination), limiting their use as unique markers.

Expanding on this work, in 2024 they applied chiral and stable isotope ratio analyses to characterize products from the same pathway, proposing the integration of isotopic profiling and organic impurity analysis as a powerful complementary tool for chemical attribution and forensic tracing of methamphetamine and ephedrine produced through emerging pathways that employ noncontrolled precursors [26].

**SCHEME 16:**
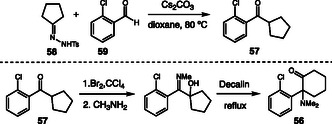
Production of ketamine.

In 2024, Yen and coworkers presented a detailed investigation into the discovery and characterization of a new synthetic route used in the illicit production of ketamine (**56**) [27]. The research originated from the seizure of a clandestine laboratory in Taiwan, where reagents, intermediates, and products related to ketamine manufacture were found. Based on the collected samples, the authors successfully reconstructed the synthetic pathway employed by the producers, demonstrating that the key precursor 2‐chlorophenyl cyclopentyl ketone (**57**) was obtained through the reaction between cyclopentanone *p*‐toluenesulfonylhydrazone (**58**) and 2‐chlorobenzaldehyde (**59**) (Scheme 16). The structure and purity of the compounds involved were determined by GC–MS, LC‐HRMS, and NMR. These analyses enabled the identification of not only the main precursor but also byproducts and residual traces that helped elucidate the reaction conditions used in the clandestine synthesis. Among the main byproducts characterized, the authors highlighted compounds formed through partial condensation or thermal decomposition of the hydrazone, such as cyclopentene derivatives and substituted hydrazones, in addition to traces of 2‐chlorobenzylidene‐cyclopentanone, which indicates an intermediate stage in the formation of the final ketone. Small amounts of hydroxylamine and a cyclic imine intermediate were also detected, suggesting parallel oxidation and rearrangement reactions.

**SCHEME 17:**
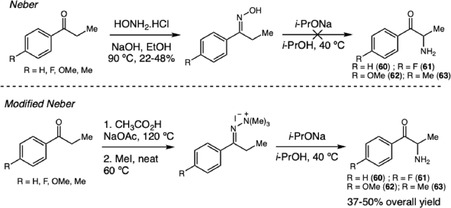
Synthesis of cathinones and derivatives.

Broadening the discussion of synthetic innovation, Loughlin and coworkers investigated the synthesis of cathinone **60** and derivatives **61–63** through the Neber and modified Neber rearrangements, aiming to assess their potential as clandestine synthetic routes, evaluate reaction viability, and identify route‐specific byproducts (Scheme 17) [28]. The classical Neber rearrangement, starting from tosyloxime propiophenones, and the modified route, employing quaternary hydrazonium salts derived from 1,1‐dimethylhydrazine and methyl iodide, both involved base‐mediated formation of 2H‐azirine intermediates, which upon aqueous acid work‐up could generate the desired cathinones. However, while the classical Neber route provided intermediates in 22%–48% yield, the subsequent oxime rearrangement predominantly yielded amides due to Beckmann rearrangement, indicating that earlier studies may have misidentified the final products as cathinones. In contrast, the modified Neber route, performed under anhydrous conditions using sodium isopropoxide, successfully afforded the target cathinone derivatives in 37%–50% overall yield, confirming it as a more viable synthetic pathway. All intermediates and final products were characterized by NMR, FTIR, GC–MS, and TOF‐HRMS and supported by DFT studies, which confirmed the transient existence of azirine intermediates and clarified mechanistic aspects of the rearrangement.

**SCHEME 18:**
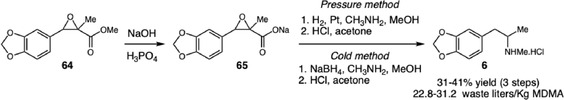
MDMA.HCl waste detection route from masked precursor.

In 2025, Laak and coworkers presented an analysis of the synthetic routes used in the illicit production of MDMA and their correlation with forensic and environmental indicators in the Netherlands, one of the main global manufacturing centers for this substance [29]. Based on data from seized clandestine laboratories and records from the Netherlands Forensic Institute, the authors described the key chemical steps involved in converting masked precursors, such as PMK methyl glycidate (**64**) and PMK glycidic acid (**65**), into PMK, and subsequently into MDMA base and MDMA.HCl (**6**) (Scheme 18). The routes evaluated include both the cold method, involving reduction with sodium borohydride, and the pressure method, using hydrogen gas and a platinum catalyst. This comparative approach allowed the estimation of yields between 34% and 41% and the generation of 22.8–31.2 L of waste per kilogram of MDMA produced, data that are fundamental for understanding the chemical processes and for tracing industrial residues associated with clandestine production. The characterization of waste, combined with yield modeling and urban wastewater monitoring, enabled the authors to estimate an annual MDMA production of 43–45 tons in the Netherlands.

**SCHEME 19:**
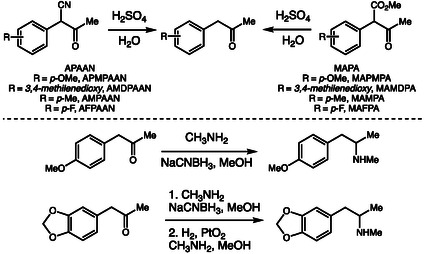
Synthesis of amphetamine‐type stimulants.

Building upon this forensic‐chemical perspective, Hägele and coworkers reported the synthesis and impurity profiling of amphetamine‐type stimulants ATS produced from ring‐substituted analogs of the precursors *α*‐phenylacetoacetonitrile (APAAN) and methyl *α*‐acetylphenylacetate (MAPA) (Scheme 19) [30]. They employed reductive amination with sodium cyanoborohydride and, in some cases, catalytic hydrogenation with platinum oxide, using precursors bearing different aromatic ring substituents. Analyses by GC–MS, HRMS, and NMR allowed the identification of specific impurities and byproducts for each route, which serve as chemical markers characteristic of the precursors and methods used.

**SCHEME 20:**
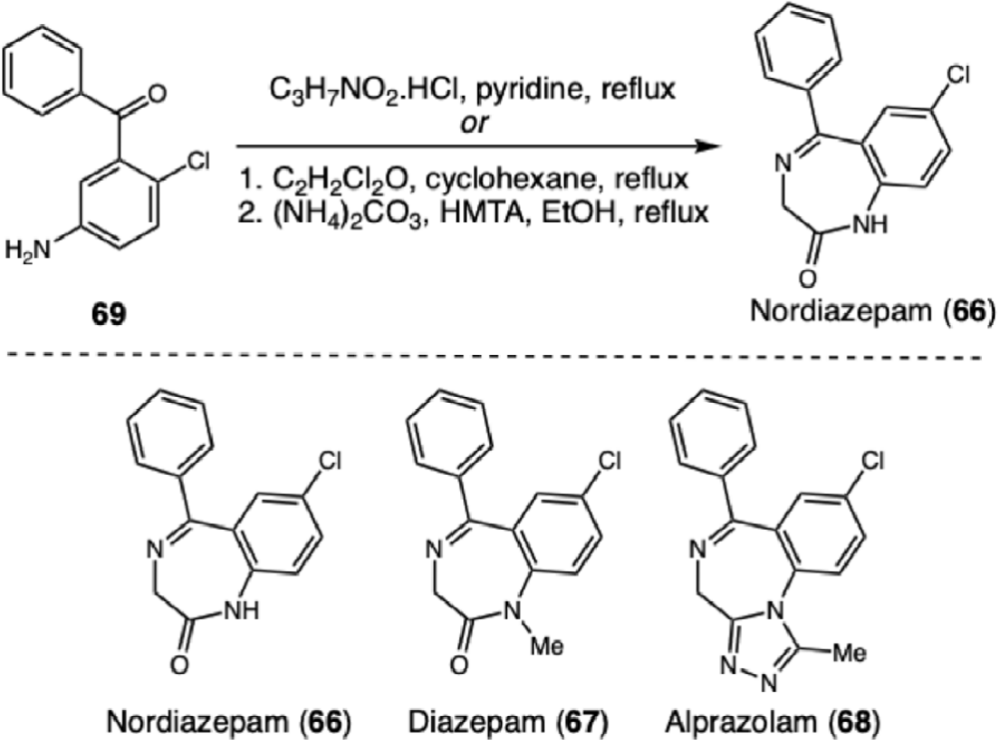
Nordiazepam synthesis.

Extending this approach to another major class of psychoactive substances, Brito and coworkers reported the investigation of alternative synthetic routes to produce nordiazepam (**66**), a key intermediate in the manufacture of benzodiazepines such as diazepam (**67**) and alprazolam (**68**) (Scheme 20) [31]. Two distinct routes were compared, both starting from 2‐amino‐5‐chlorobenzophenone (**69**). The routes yielded products with purities ranging from 50% to 95%, and detailed analyses by GCxGC‐TOFMS enabled the identification of 34 characteristic impurities and byproducts, which act as chemical markers specific to each route and constitute the set of impurities known as the Chemical Attribution Signature (CAS).

## 3 Forensic Toxicology

Toxicology plays a fundamental role in forensic science, providing essential information for the detection and identification of chemical substances in biological samples, contributing to the elucidation of cases involving death, intoxication, or drug exposure [32]. From a metabolic perspective, after drug administration, xenobiotic compounds undergo phase I biotransformation reactions, including oxidation, reduction, and hydrolysis, mainly catalyzed by cytochrome P450 enzymes. These reactions result in minor structural modifications through the introduction or exposure of functional groups, increasing the reactivity and enabling subsequent phase II metabolism. Phase II enzymes catalyze conjugation reactions with endogenous polar moieties, such as glucuronic acid, leading to highly hydrophilic phase II metabolites that are more readily excreted in urine [33].

Over the past decades, the deeper understanding of metabolism and the integration in forensic science has shown remarkable progress, driven by the rigorous validation of analytical methods and the incorporation of high‐resolution instrumental techniques, which have significantly enhanced the sensitivity, selectivity, and reliability of toxicological analyses [34]. These advancements have enabled the accurate detection of compounds in various biological matrices, such as blood, urine, hair, and oral fluid, strengthening the quality and robustness of forensic interpretations [35]. At the same time, the continuous emergence of new psychoactive substances has posed growing analytical and legal challenges, demanding innovative approaches and the development of synthesized reference standards for structural confirmation and method validation. In this context, the Forensic Toxicology section of this review is organized into two main topics: new substances and metabolic studies, centered on the characterization of NPS and their metabolites, and human performance toxicology, focused on evaluating the metabolites through the synthesis of reference standards.

### 3.1 New Substances and Metabolic Studies

**SCHEME 21:**
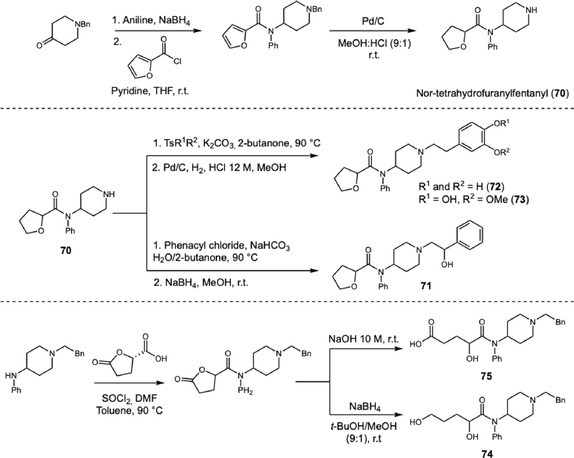
Synthesis of THF‐fentanyl metabolites (**71–75**) identified by LC‐MS after incubation with fresh human hepatocytes.

Over the years, novel synthetic opioids have continued to emerge in the illicit drug market. Tetrahydrofuranylfentanyl (THF‐Fentanyl) appeared in 2015 in the United States and has been associated with fatal intoxications and multiple seizures. In 2020, Kanamori and coworkers [36] investigated the metabolism of THF‐Fentanyl by incubation with fresh human hepatocytes (FHH). LC‐MS analysis enabled the identification of the nonmodified drug together with 12 phase I and phase II metabolites (Scheme 21). The main metabolic pathways involved cleavage of the phenylethyl moiety (**70**), and formation of several hydroxylated metabolites at the piperidine ring (**71**), and oxidation at the phenylethyl side chain (**72, 73**). In addition to hydroxylation, the furanyl ring underwent oxidative cleavage, yielding ring‐opened alcohol (**74**) and ring‐opened carboxylic acids (**75**), sometimes in combination with modifications at other sites. Several phase II metabolites were identified, most of them as glucuronides of phenylethyl hydroxylated derivatives. Among all metabolites, **75** showed the highest peak intensity, suggesting high abundance and its potential as an important biomarker for THF‐fentanyl identification.

To confirm the metabolite structures, the authors synthesized reference standards beginning with the reductive amination of *N*‐protected piperidone, followed by coupling with furoyl chloride to afford **70** after nitrogen deprotection. This intermediate was subsequently alkylated with various tosylates or phenacyl chloride to yield the corresponding side‐chain‐oxidized derivatives. Finally, the ring‐opening alcohol and carboxylic acid metabolites were obtained through cleavage of the tetrahydrofuranyl side chain under basic conditions or by reduction, respectively. The major metabolites formed in FHH incubations were then confirmed by comparison of retention times and MS fragmentation patterns with the synthesized standards.

**SCHEME 22:**
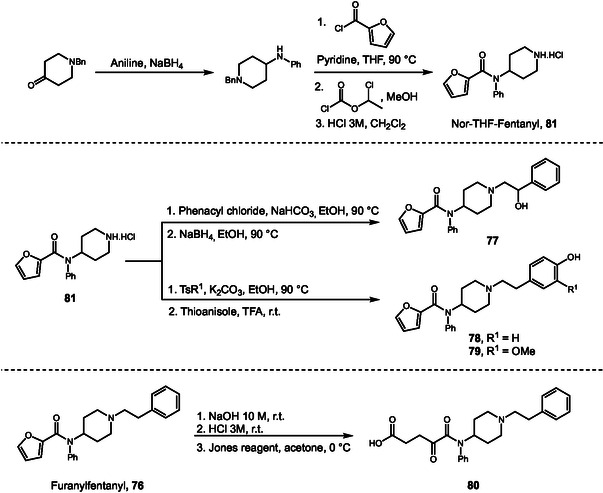
Synthesis of furanylfentanyl metabolites (**77–81**) identified by LC‐MS after incubation with fresh human hepatocytes. Major metabolic transformations occurred at the phenylethyl moiety, along with ring opening of the furanyl ring.

The continuous emergence of novel fentanyl derivatives is driven by slight structural modifications of the parent drug, which can yield new substances with enhanced potency. Since 2015, a more potent analog, furanylfentanyl (**76**), has gained popularity in the USA and Europe, with approximately 500 reported cases of acute intoxication. However, current knowledge regarding its metabolism remains limited, hindering its reliable detection in biological matrices. In 2021, Kanamori and coworkers [37] investigated the metabolism of **76** by incubation with fresh human hepatocytes. LC‐MS analysis of the culture medium revealed the parent drug and four metabolites arising from oxidation of the phenylethyl moiety (**77–79**) and ring opening of the furanyl group to a carboxylic acid derivative (**80**), with **80** presenting the highest peak intensity, indicating that it is the major metabolite. In contrast to previous in vivo reports, the authors did not detect nor‐furanylfentanyl (**81**), formed by cleavage of the phenylethyl moiety, suggesting the absence or low activity of a specific amidase in FHH. Given the structural similarity to tetrahydrofuranylfentanyl [36], the authors employed a similar synthetic route to prepare the reference standards, only by replacing the acyl chloride for furoyl chloride. The structures of metabolites **77–81** were confirmed through comparison of retention times and MS data with the synthesized authentic standards (Scheme 22).

**SCHEME 23:**
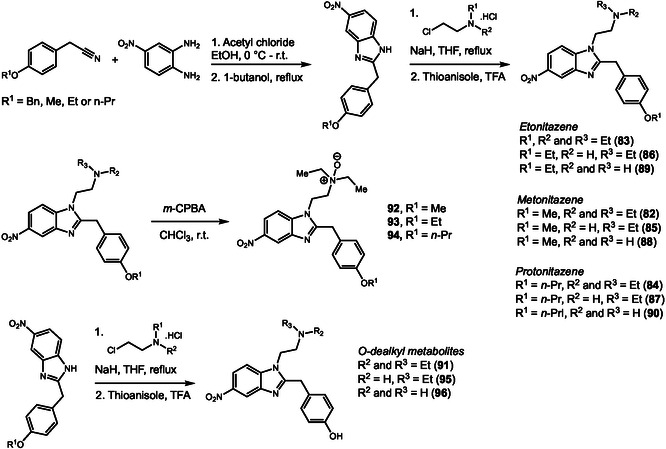
Synthesis of nitazene and nitazene metabolites identified after incubation with fresh human hepatocytes.

After the strict regulation of fentanyl and its analogs, a new class of synthetic opioids emerged in the illicit drug market. Nitazenes, which contain a benzimidazole core and were originally developed as potent analgesics, have since gained popularity as drugs of abuse. Despite the increasing number of reports on nitazenes in recent years, knowledge of their metabolism remains limited, with most studies relying solely on mass spectrometry data. In 2025, Kanamori and coworkers [38] investigated the metabolism of the nitazene analogs metonitazene (**82**), etonitazene (**83**), and protonitazene (**84**) by incubation with FHH (Scheme 23). LC‐MS analysis of the culture medium demonstrated that all three nitazenes displayed a similar metabolic profile, characterized by *N*‐dealkylation (**85**, **86**, and **87**), *N*‐didealkylation (**88**, **89**, **90**), *O*‐dealkylation (**91**), *N*‐oxidation (**92–94**), and combined *N*‐ and *O*‐dealkylation (**95, 96**). Several phase II metabolites were also detected, mainly as glucuronide conjugates. MS fragmentation of one phase II metabolite revealed an atypical oxidation at the 1‐position of the *N*‐ethyl. Although oxidation at this site typically results in dealkylation, subsequent conjugation yielded a stable metabolite. To confirm the structures of the major metabolites the authors synthesized authentic standards beginning with construction of the benzimidazole core followed by *N*‐alkylation using different alkyl chlorides. Additionally, the parent drugs were oxidized using *m*‐chloroperbenzoic acid (*m*‐CPBA), to obtain the *N*‐oxide metabolites. After synthesis, retention times and MS data of the standards were compared with those of the metabolic products, to confirm the structure of the detected metabolites.

Taken together, these findings highlight how the synthesis of reference standards combined with the analysis of biological matrices has been essential for unraveling the complex metabolic pathways of structurally diverse drugs. Building on this approach, other studies have expanded this metabolic landscape by investigating compounds from other types of NPS, as cathinones. In 2020, Carrola and coworkers [39] investigated the metabolism of the new psychoactive substances *N*‐ethylhexedrone (NEH) (**97**) and buphedrone (BUH) (**98**) using HPLC‐MS to identify and quantify the main phase I and phase II metabolites excreted in mouse urine after 24 h of drug administration. LC‐MS analysis of urine samples showed that the parent compounds were excreted at low levels (<10%) and both drugs exhibited similar metabolic patterns. The major metabolites resulted from *N*‐dealkylation H2 (**99**) and B2 (**100**), followed by products combining *β*‐keto reduction and *N*‐dealkylation H3 (**101**) and B3 (**102**). Additional minor metabolites were detected, including *β*‐keto‐reduced N‐alkylated forms H1 (**103**) and B1 (**104**) and B4 an aromatic 4‐hydroxylated metabolite (**105**), observed only for buphedrone. Phase II metabolites were also identified, formed through reactions such as *N*‐acetylation, glucuronidation, and further conjugation of the *N*‐dealkylated metabolite with succinic, glutaric, or adipic acids.

**SCHEME 24:**
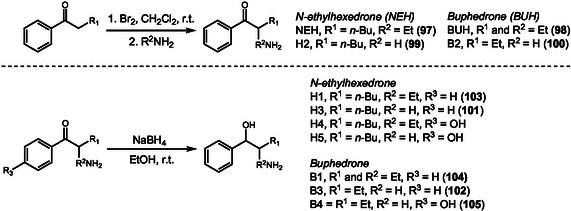
Synthesis of buphedrone and *N*‐ethylhexedrone metabolites identified in mouse urine by LC‐MS analysis after administration of parent drugs in mice.

The parent drugs and selected metabolites H1‐H5 and B1‐B4 were synthesized (Scheme 24). To obtain the target compounds, the corresponding ketones were first treated with molecular bromine to form the *α*‐bromoketone intermediates. These bromine intermediaries undergo nucleophilic substitution with various amines, such as ammonia, methylamine, or ethylamine, to afford the desired *α*‐aminoketones. In addition, the keto‐reduced metabolites were prepared by reducing the parent ketones with NaBH_4_. The resulting reference standards were then used to confirm metabolite structures detected in mouse urine by comparing retention times and MS fragmentation patterns.

**SCHEME 25:**
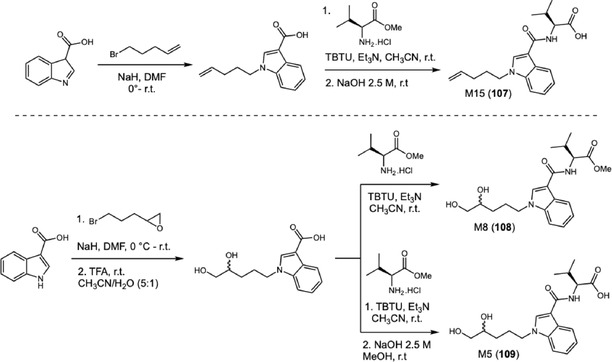
MMB022 metabolites identified from pentenyl side‐chain oxidation (M5 and M8) and from ester hydrolysis (M16), with their structures confirmed by synthesis of authentic standards.

Subsequent studies have focused on synthetic cannabinoids, particularly those bearing uncommon functional groups that may influence metabolic pathways. In 2020, Watanabe and coworkers reported advances in understanding the metabolism of the synthetic cannabinoid MMB022 (**106**), a NPS recently identified in the European market and notable for presenting a terminal olefin in the pentenyl side chain, a rare structural modification among synthetic cannabinoids (Scheme 25) [40]. To elucidate its main metabolic processes, the authors synthesized reference standards for the predicted metabolites, including diol derivatives, intermediate epoxides, and ester hydrolysis products, all of which were characterized by NMR spectroscopy and LC‐QTOF‐MS. Incubation of **106** with human liver microsomes revealed the formation of sixteen phase I metabolites, mainly resulting from diol formation, dehydrogenation, ester hydrolysis, and hydroxylation, confirming that dihydroxylation of the alkene group represents the predominant biotransformation pathway. This reaction likely proceeds via a cytochrome P450‐mediated mechanism, involving the transient generation of epoxide intermediates that are subsequently converted by epoxide hydrolases into more polar and biologically excretable compounds. Among the identified metabolites, M15 (**107**), M8 (**108**), and M5 (**109**) were the most abundant, with M5 characterized as the final stable product of the metabolic cascade and therefore a potential predominant metabolite under physiological conditions. The metabolites were synthesized through *N*‐alkylation of 3‐indolyl carboxylic acid with various alkyl bromides, followed by coupling with valine methyl ester to obtain the amide‐linker. M15 was obtained after methyl ester hydrolysis. For the dihydrodiol derivatives, the epoxide side chain was subjected to acid‐mediated hydrolysis to yield M8, and subsequent methyl ester hydrolysis to obtain M5.

Metabolic stability studies demonstrated that **106** exhibits an extremely short in vitro half‐life and a rapid biotransformation. These findings corroborate previous observations regarding the pharmacokinetic behavior of synthetic cannabinoids, which tend to undergo extensive hepatic metabolism and show low detectability of the unchanged parent compound in biological samples.

### 3.2 Human Performance Toxicology

**SCHEME 26:**
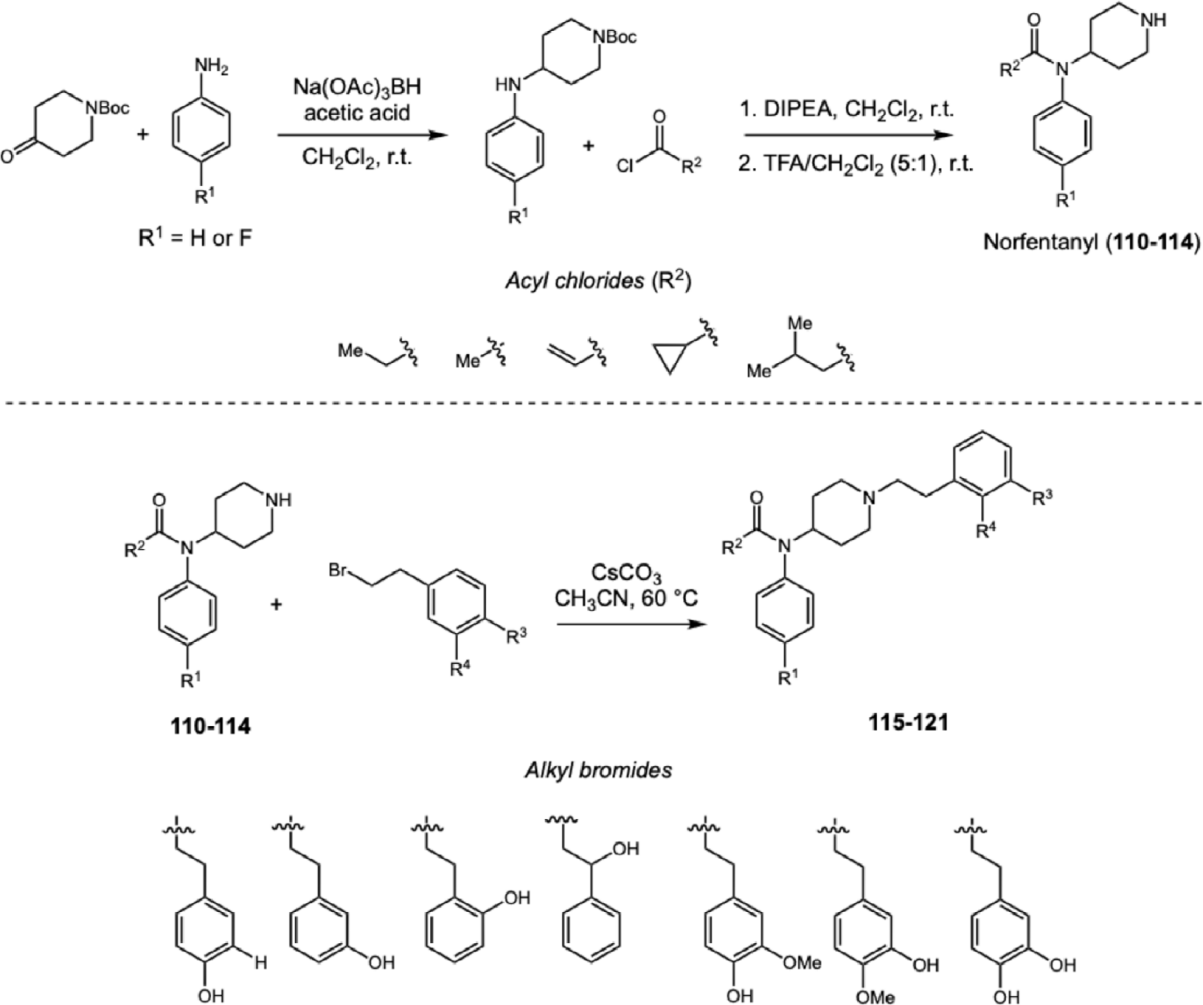
Synthetic strategy to obtain nor‐fentanyl derivatives (**110**‐**114**) and the main metabolites formed through CYP450‐catalyzed oxidation of the phenylethyl moiety.

Fentanyl and its analogs represent a highly dangerous group of new psychoactive substances, responsible for numerous fatal intoxications worldwide. Knowledge of their metabolism is still limited, as authentic reference standards for metabolite confirmation are often unavailable. In 2020, Wallgren and coworkers investigated the metabolism of fentanyl and five analogs, acetylfentanyl, acrylfentanyl, cyclopropylfentanyl, isobutyrylfentanyl, and 4‐F‐isobutyrylfentanyl using human liver microsomes (HLM) incubation (Scheme 26) [41]. LC‐QTOF‐MS analysis of the incubated samples revealed that the metabolic patterns of these fentanyls are similar, predominantly involving CYP450‐mediated hydroxylation at the 4‐ and *β*‐positions, and the formation of catechol‐like metabolites with a 4‐OH‐3‐OMe substitution pattern, at the phenylethyl moiety. The *β*‐hydroxylated metabolite was the most abundant in five out of six fentanyl derivatives, with exception of acetylfentanyl where 4‐OH was more abundant. The abundance of *β*‐OH decreased proportionally with increasing of amide side‐chain length. Additionally, in authentic urine samples, other catechol‐like metabolites 3‐OH‐4‐OMe and 3,4‐diOH were detected. Among them, oxidation at position 4′‐OH was the most abundant metabolite overall the fentanyl analogs. The metabolite structures were confirmed by in‐house synthesis of reference standards followed by comparison of retention times and mass fragmentation profiles. To obtain this wide diversity of standards, the authors proposed a concise synthetic route beginning with the reductive amination of *N*‐Boc‐piperidone with aniline or 4‐F‐aniline. The resulting amines were then acylated with different acyl chlorides, yielding the corresponding norfentanyl analogs (**110–114**). After removal of the nitrogen protecting group, the piperidine nitrogen was alkylated with various alkyl bromines, enabling the preparation of fentanyl analogs bearing distinct oxidation patterns at phenylethyl side chain (**115–121**).

After confirming the structures of the main metabolites by comparison of the retention times and MS data, the authors also observed differences between the metabolite profiles in urine samples and HLM, which may be related to several factors, such as accumulation during urine formation, concomitant drug use, or genetic variations in CYP isoforms. Further studies are necessary to clarify the discrepancies between the metabolites detected in urine and those generated by HLM incubation.

Synthetic cathinones (SCs) have been available on the market since the 1920s. In recent years, there has been a growing increase in the number of cathinone‐based drugs, which are often illegally sold on the internet and abused worldwide [42]. Given the structural diversity within this class and the toxicological relevance associated with their widespread use, recent studies have focused on elucidating the metabolic pathways of emerging SCs to improve detection and understand their metabolism for further forensic application.

**SCHEME 27:**
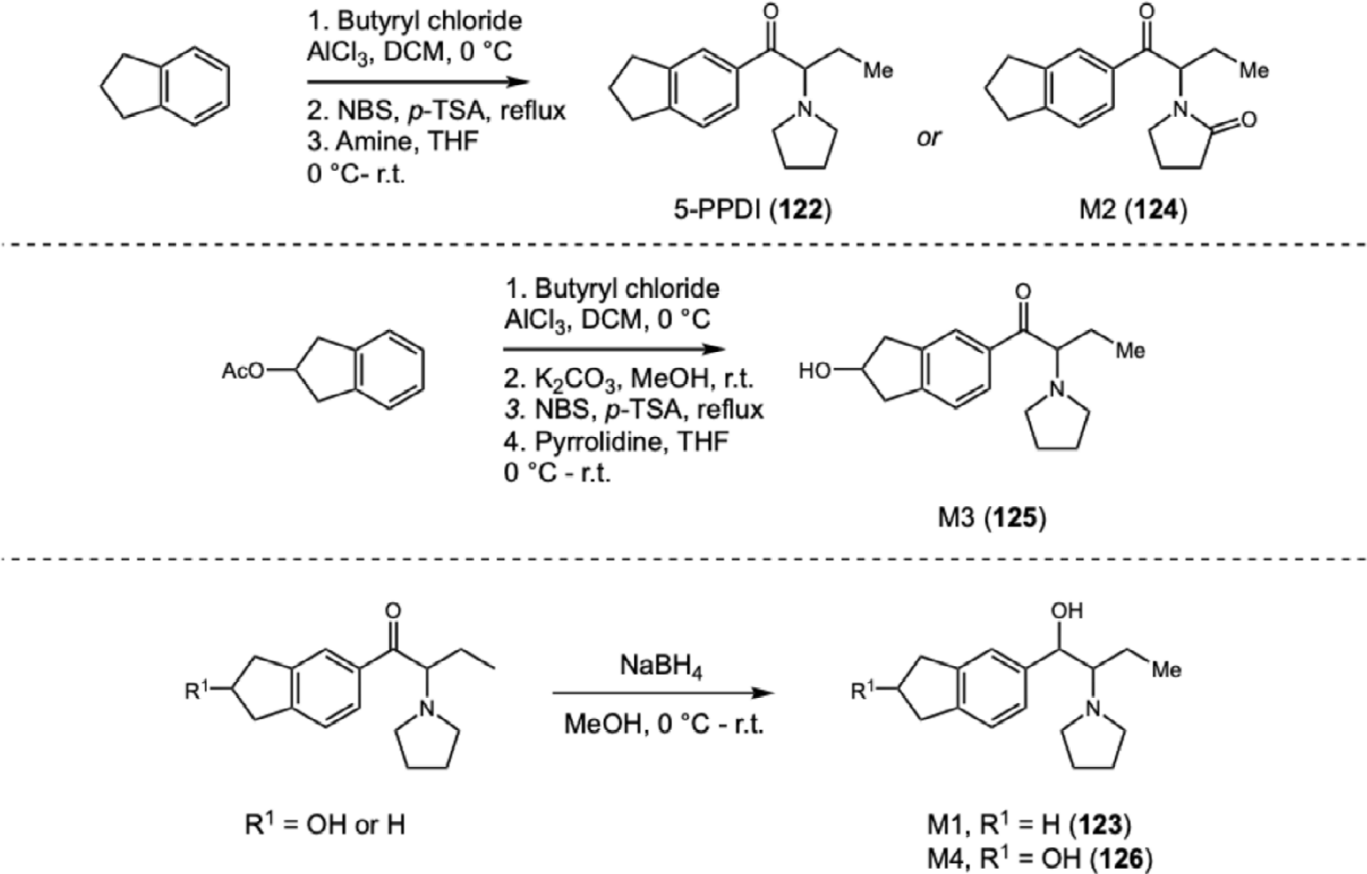
Synthesis of 5‐PPDI metabolites M1‐M4.

In 2020, Ishii and coworkers investigated the metabolism of the synthetic cathinone 5‐PPDI (**122**), a NPS belonging to the pyrrolidinophenone class (PP), compounds detected in seizures and forensic cases related to the consumption of emerging psychoactive substances (Scheme 27) [43]. This compound is structurally distinguished from other cathinones by the presence of an indane ring in its aromatic portion, which can significantly influence its metabolic pathways and pharmacokinetic properties. To elucidate these characteristics, four reference metabolites **123–126** were synthesized and characterized by NMR spectroscopy and LC‐MS. 5‐PPDI metabolites were synthesized via Friedel–Crafts acylation of indane or *O*‐protected indane, catalyzed by aluminum(III) chloride. The resulting ketone was then *α*‐brominated, and the intermediate bromide was reacted with pyrrolidine to afford 5‐PPDI and **125**, or with pyrrolidinone to obtain **124**. The *α*‐amino ketones **125** and **122** were subsequently reduced to yield the keto‐reduced metabolites **123** and **126**.

Through the analysis of human urine samples, the researchers identified 16 phase I metabolites, demonstrating that **122** undergoes extensive and diverse metabolic transformations. The main biotransformation pathways included hydroxylation, oxidation of the pyrrolidine ring, and stereoselective reduction of the carbonyl group, observed for the first time in PP derivatives in humans, representing a novel finding in toxicological literature. The results also revealed that hydroxylation of the indane ring is the predominant metabolic route, leading mainly to the formation of the **125**, which was identified as the most abundant product in the analyzed samples, followed by **126**. This predominance suggests that the indane ring is highly susceptible to oxidation catalyzed by cytochrome P450 enzymes, which are responsible for introducing hydroxyl groups into xenobiotic compounds.

Immunoassays are widely used in routine toxicological screening. However, the emergence of novel NPS with structural similarities to known drugs can lead to cross‐reactivity. Several studies have reported cross‐reactivity of synthetic cathinones in assays targeting amphetamine‐type substances. To address this limitation, forensic laboratories have increasingly adopted fast and robust LC‐MS methods for the identification and quantification of cathinones in biological samples.

**SCHEME 28:**
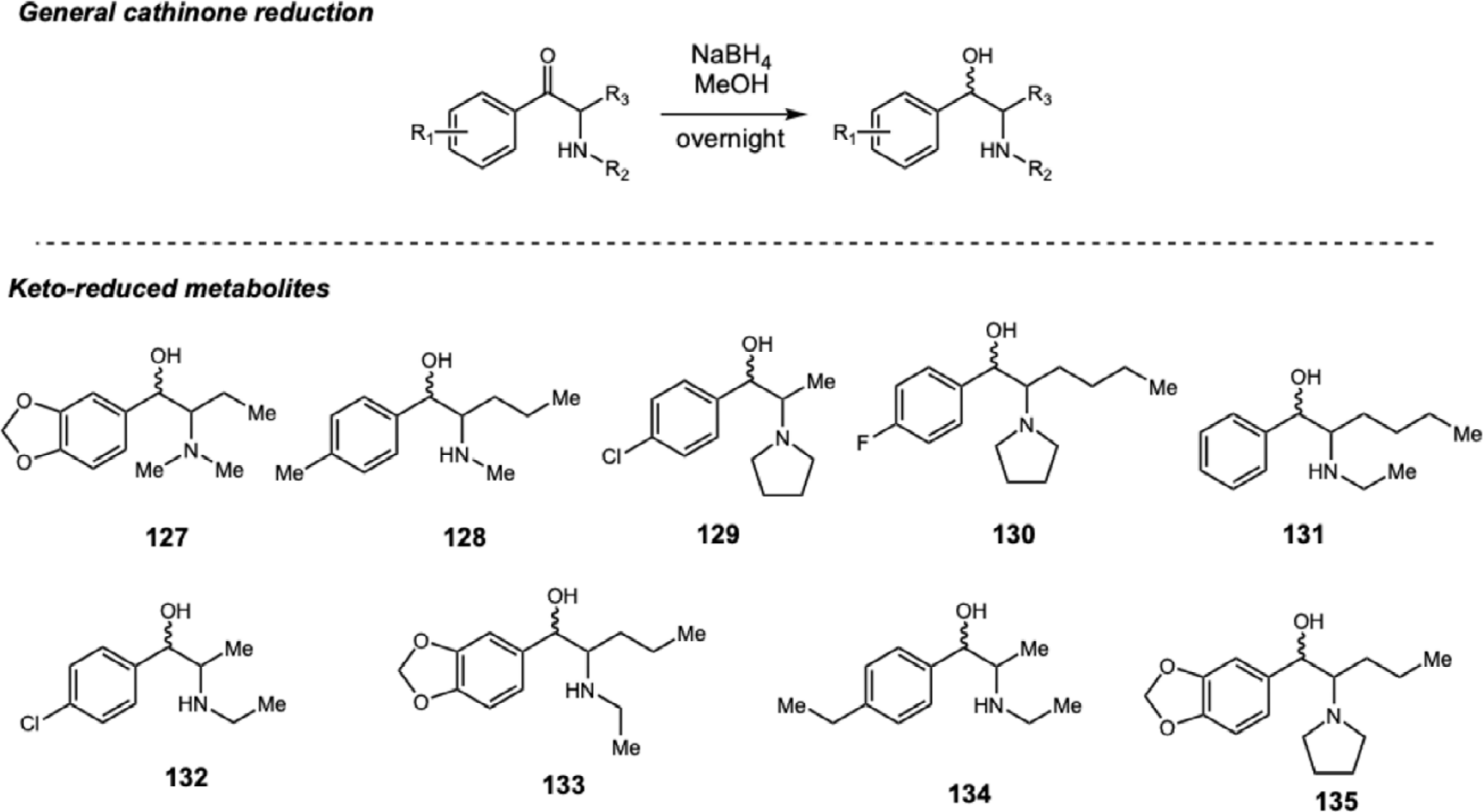
Keto‐reduction of a series of SCs for doping analysis in urine samples from Saudi Arabia.

In 2022, Aldubayyan and coworkers developed an LC‐MS method for detecting and quantifying 16 synthetic cathinones (SCs) and 10 metabolites in suspicious urine samples from Saudi Arabia (Scheme 28) [44]. These samples had initially tested positive for amphetamines in immunoassay screening but were negative in confirmatory analyses, suggesting possible cross‐reactivity with cathinones due to structural similarities. Because no data on SC consumption were available for Saudi Arabia, the authors selected the most prevalent SCs reported worldwide, including dibutylone, 4‐chloroethcathinone (4‐CEC), 4‐chloro‐*α*‐pyrrolidinopropiophenone (4‐Cl‐*α*‐PPP), 4‐ethylmethcathinone (4‐EMC), *N*‐ethylpentylone, methylenedioxypyrovalerone (MDPV), 4‐methylpentedrone (4‐MPD), *N*‐ethylhexedrone, and 4‐fluoro‐*α*‐pyrrolidinohexanophenone (4‐F‐PHP). The chromatographic method was validated according to international guidelines, and the reference standard of the keto‐reduced metabolites (**127–135**) was synthesized via NaBH_4_‐mediated reduction of the parent drugs. A total of 52 real‐case urine samples were analyzed, of which 5.8% were positive for at least one cathinone. In all positive cases, the reduced metabolites were detected alongside the parent compounds.

Stability of drugs and its metabolites is an important parameter during doping analysis. In this context, in 2024 Aldubayann and coworkers [45] evaluated the short‐ and long‐term stability of SCs and keto‐reduced metabolites in urine samples. Beyond the SCs and keto‐reduced metabolites synthesized in previous studies by the group [44], butylone, ethylone, methedrone, methylone, *α*‐pyrrolidinovalerophenone (*α‐PVP*), and 4‐chloro‐pyrrolidinovalerophenone (4‐Cl‐*α*‐PVP) and the corresponding keto‐reduced metabolites were included in the study. Stock urine samples were prepared by spiking drug‐free human urine with each parent compound or keto‐reduced metabolite. These samples were stored for 3 and 14 days at room temperature and 4°C to assess short‐term stability, and at −40°C for 1 year to assess long‐term stability. All samples were analyzed by LC‐MS to quantify each drug or metabolite after the predetermined storage periods.

At short‐term storage under both temperatures, most samples presented stability issues, with a significant decrease in concentration. 4‐CEC exhibited the most pronounced reduction, with less than 20% of the initial concentration remaining, while the other cathinones retained around 40% at room temperature and 50% at 4°C. In contrast to the parent drugs, most metabolites, except for the halogenated ones, remained stable (>60% remaining) after 14 days at both temperatures. Regarding long‐term storage, most parent drugs and metabolites were stable under the tested conditions, with overall losses below 25%, except for the halogenated analytes. After 6 and 12 months, the concentrations were maintained within this range. Overall, the authors concluded that storage temperature and the presence of halogen substituents directly influence the stability of SCs by accelerating their degradation. Conversely, the keto‐reduced metabolites showed marked stability under all storage conditions, when compared with the parent drugs.

**SCHEME 29:**
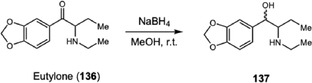
Synthesis of the eutylone metabolite **137** obtained by NaBH_4_‐mediated reduction of the parent drug.

Considering the challenges associated with the stability and detection of SCs and their metabolites, subsequent investigations have focused not only on their behavior in biological matrices but also on improving the identification of these compounds during routine forensic and doping analyses. In 2024, Pelletier and coworkers [46] were requested to perform a doping analysis on blood samples from two drug users arrested by the police. Along with the couple, a suspicious white powder was also seized, which was presumed to contain eutylone (**136**) (Scheme 29). LC‐HRMS and NMR analyses confirmed the presence of eutylone in the seized sample, with a high purity. Using LC‐HRMS data, the authors applied chemoinformatic tools to identify potential eutylone metabolites in the blood samples. Molecular networking enabled the annotation of several phase I metabolites, mainly resulting from mono‐ and di‐demethylation, as well as hydroxylation and *β*‐keto reduction (**137**). To increase confidence in the identification of **137** and allow its quantification in biological matrices, the metabolite was synthesized via reduction of the parent compound. Comparison of retention times and MS data confirmed the presence of eutylone and **137** in the blood samples, with concentrations up to 1300 and 50 ng mL^−1^, respectively.

**SCHEME 30:**
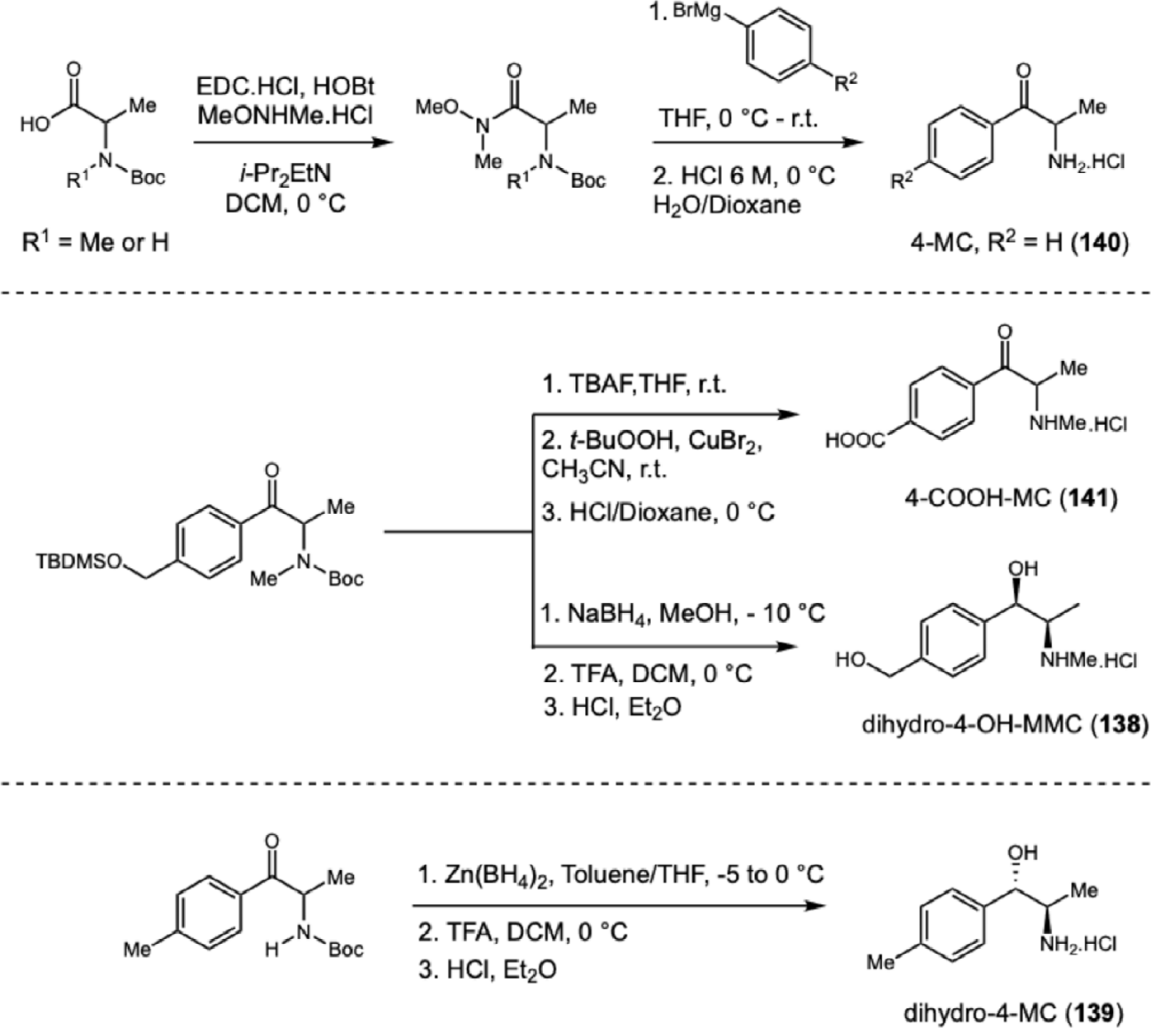
Synthesis of mephedrone metabolites identified in urine samples.

Beyond advancing the structural and metabolic characterization of SCs, these findings also reinforced the importance of integrating metabolic profiling with functional assessment when evaluating the toxicological relevance of emerging cathinones. This broader perspective has driven subsequent investigations toward not only identifying metabolites but also understanding their potential pharmacological impact. In 2021, Niello and coworkers investigated the pharmacological potential of mephedrone (MPH), a potent new psychoactive substance (NPS) with effects similar to those of ecstasy (Scheme 30) [47]. The modulation of the nervous system is attributed not only to MPH itself but also to its metabolites, which can interact with serotonin (SERT), dopamine (DAT), and norepinephrine (NET) transporters. MPH undergoes stereospecific metabolism by CYP2D6, yielding metabolites formed through *N*‐dealkylation, benzylic oxidation, *β*‐keto reduction, or combinations thereof. Moreover, the enantiomers of MPH may exhibit distinct biological activities. To explore these differences, the authors synthesized enantiopure MPH metabolites starting from D/L‐alanine. The keto‐reduced metabolites di‐hydro‐4‐OH‐MMC (**138**) and di‐hydro‐4‐MC (**139**) were obtained by stereoselective carbonyl reduction using either NaBH_4_ or Zn(BH_4_)_2_. In vitro assays at DAT, NET, and SERT transporters showed that the metabolites exhibited negligible inhibition at SERT, with only one metabolite displaying comparable potency to MPH at DAT and NET. In addition to pharmacological evaluation, the synthesized metabolites were used as reference standards to confirm MPH consumption in urine samples by LC‐MS. Using an achiral method, the authors identified 4‐OH‐MMC, 4‐MC (**140**), 4‐COOH‐MC (**141**), and both anti‐ and syn‐dihydro‐4‐MC in a 1:1 ratio. In contrast to previous reports, metabolites derived only from *β*‐keto reduction were not detected.

Amphetamine and methamphetamine are widely known as potent central nervous system stimulants. Recently, novel derivatives with enhanced activity have appeared on the illicit drug market, fluoromethamphetamine (FMA) and fluoroamphetamine (FAP). To date, different regioisomers of FMA and FAP, distinguished based on the position of the fluorine atom on the aromatic ring, have been identified by EMCDDA. However, knowledge about the metabolism of these drugs remains limited. In 2023, Ishii and coworkers were requested to analyze a urine sample from a drug user as part of a criminal investigation to identify metabolites associated with the consumption of 2‐fluoromethamphetamine (2‐FMA) and 2‐fluoroamphetamine (2‐FAP) [48]. HPLC‐MS analysis revealed metabolites formed by aromatic hydroxylation, aliphatic hydroxylation (**142**), and *N*‐hydroxylation (**143**). The metabolism of amphetamine‐like drugs is well established, occurring mainly by phase I reaction catalyzed by CYP2D6 isoform.

**SCHEME 31:**
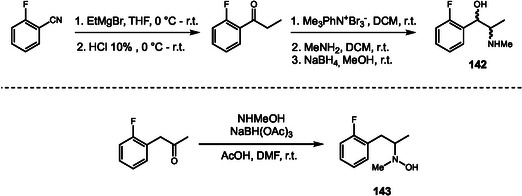
Synthesis of 2‐FMA metabolites, *N*‐hydroxy (**143**) and ketoreduced (**142**).

To confirm the structures of the major metabolites, the authors tried to synthesize authentic standards (Scheme 31). Metabolite **142** was synthesized in four consecutive steps: a one‐pot Grignard addition to the corresponding nitrile and acid‐mediated hydrolysis of the intermediate imine, *α*‐bromination of the resulting ketone, substitution of the bromide by methylamine, and a final carbonyl reduction. In turn, metabolite **143** was successfully obtained by reductive amination of the parent ketone using NaBH(OAc)_3_ as the reducing agent. The authors also attempted to prepare ring‐hydroxylated metabolites; however, these compounds could not be obtained. Based on the synthesized standards, the authors performed qualitative and quantitative analyses to confirm 2‐FMA intake and highlighted that the drug is primarily excreted as **142** and **143**.

Synthetic cannabinoid receptor agonists are a chemically diverse group of new psychoactive substances that exert their effects on the endocannabinoid system by the activation of CB1 and CB2 receptors [49], triggering a wide range of actions (e.g., elevated mood, relaxation, appetite stimulation) similar to those induced by cannabis [50]. However, over the years, more than 224 SCRAs have been reported by EMCDDA and a progressive increase in intoxication and death cases, representing a major public health concern. To date, knowledge regarding the metabolism and stability of these drugs remains limited, highlighting the need to identify their main metabolic pathways and reliable biomarkers for doping analysis.

**SCHEME 32:**
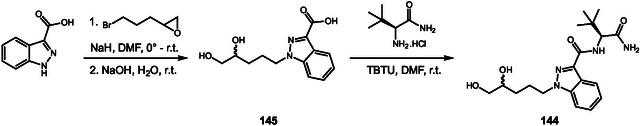
Synthesis of dihydrodiol metabolite (**145**) from pentenyl side‐chain oxidation of ADB‐4en‐PINACA.

In 2021, Kronstrand and coworkers investigated the metabolism of ADB‐BUTINACA and ADB‐4en‐PINACA, two indazole‐type synthetic cannabinoids frequently detected in seizures across Europe after 2019 (Scheme 32) [49]. The compounds share the same core and linker structure, differing only by the size and the presence of a terminal double bond at the tail at ADB‐4en‐PINACA. In vitro metabolism studies using HLM were conducted and LC‐MS analysis showed that both substances underwent similar metabolic pathways, suffering mainly hydrolysis, dehydrogenation, dihydrodiol formation, hydroxylation, and combinations of these transformations. For ADB‐BUTINACA, the most abundant metabolites arose from hydroxylation of either the indazole ring or the side chain. In addition to in vitro data, urinary analysis confirmed the presence of monohydroxylated metabolites B8, B9, and B16. However, the hydroxylation position at B8 and B9 could not be confirmed based only on MS data. In contrast, ADB‐4en‐PINACA was primarily metabolized at the double bond of pentenyl chain, forming a dihydrodiol metabolite (**144**) via an epoxide intermediate. To confirm the structure of the main metabolite, the authors synthesized **145** through an *N*1‐indazole carboxylic acid alkylation using an alkyl bromide bearing an epoxide side chain, which was subsequently opened under basic hydrolysis. The resulting intermediate was then coupled with *tert*‐leucinamide, yielding **144**.

**SCHEME 33:**
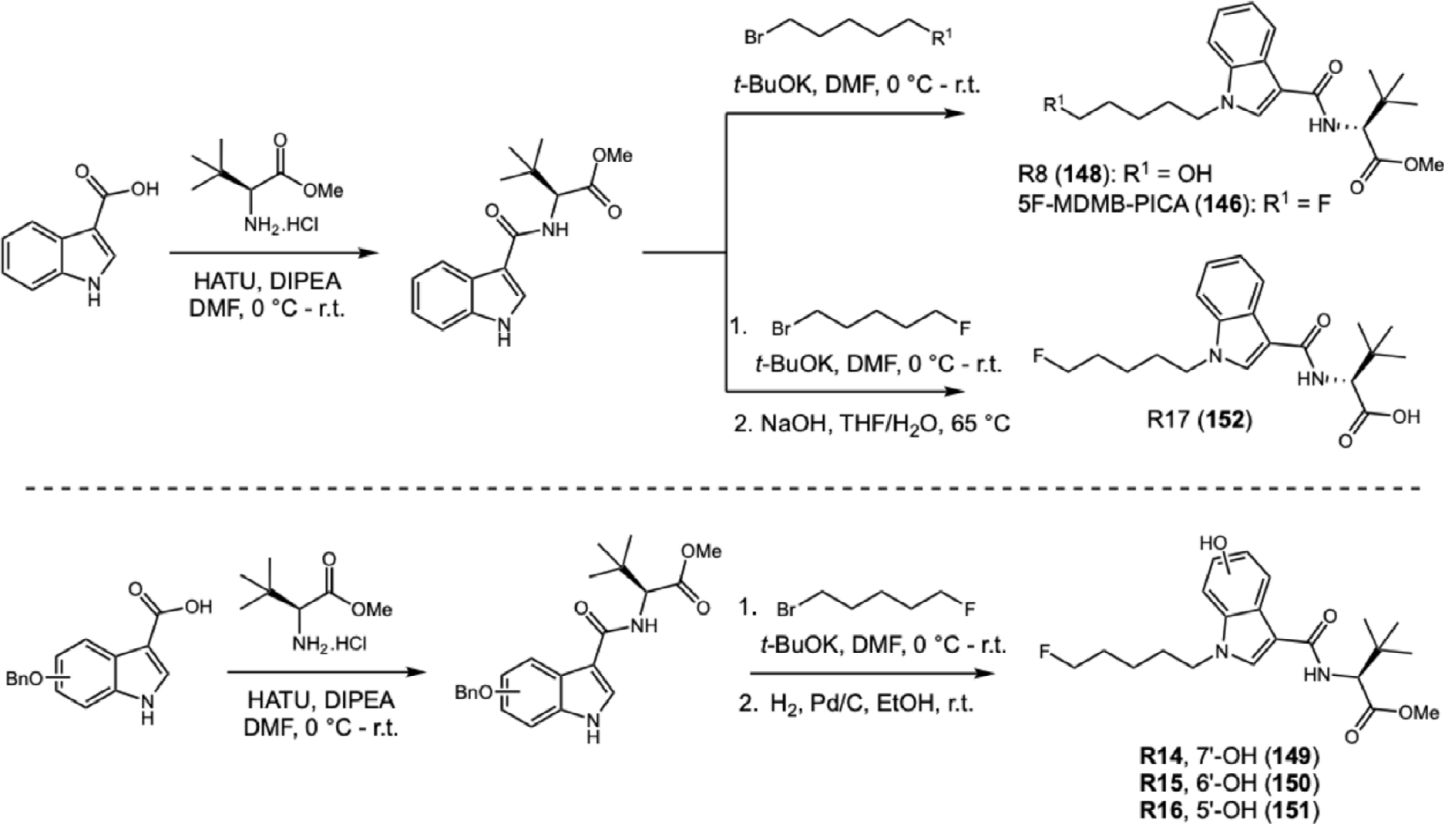
Scheme caption. Synthesis of 5F‐MDMB‐PICA metabolites R8 and R14‐R17.

Together, these findings illustrate the diversity of metabolic pathways observed across different subclasses of SCRAs. Building on this metabolic framework, subsequent studies have focused on other structurally distinct cannabinoids, such as 5F‐MDMB‐PICA (**146**), a novel indole‐type synthetic cannabinoid. In 2024, Chen and coworkers investigated the main metabolites of 5F‐MDMB‐PICA (**146**), a compound with strong affinity for the CB_1_ receptor, significantly more potent than THC (Scheme 33) [51]. Although several studies had previously examined its metabolism, the structures of the major metabolites could not be conclusively established without reference standards. To address this, the authors synthesized 17 potential phase I metabolite standards and performed systematic characterization by NMR, FT‐IR, and HRMS. According to the literature, the main metabolites of indole‐type SCRAs resulted from hydrolysis of the tert‐leucine amide (**147**) or methyl ester (**146** or **148**), aromatic oxidation (**149–151**), side‐chain oxidation, or N‐dealkylation. LC‐MS/MS analysis of an authentic urine sample from an adult user revealed that the ester‐hydrolyzed metabolite **152** was the most abundant, while metabolites formed through side‐chain oxidation (**148**) and aromatic oxidation (**149–151**) were present at lower levels. The metabolites identified in urine analysis were confirmed by the synthesis of authentic samples. The authors adopted a concise synthetic route to obtain the desired metabolites, beginning with the coupling of 3‐indolecarboxylic acid with *t*‐leucine methyl ester. The resulting carbamate was then alkylated with the corresponding alkyl bromide, affording compound **148**. Finally, base‐mediated hydrolysis of the methyl ester yielded **152**. For metabolites **149–151**, *O*‐protected indolyl carboxylic acids were used as starting materials and subjected to the same coupling and alkylation steps, followed by deprotection to afford the 7′‐ (R14), 6′‐ (R15), and 5′‐hydroxy (R16) derivatives.

Beyond SCRAs, novel semisynthetic cannabinoids have appeared in liquid cartridges for electronic cigarettes and herbal products. Their pharmacological effects are similar to those of THC, exhibiting potent psychotropic activity [52]. Hexahydrocannabinol (HHC) has been circulating in Japan since at least 2022, where its consumption is subject to legal penalties [53]. Several metabolites of HHC have been detected in urine samples, most notably 11‐nor‐9‐carboxy‐hexahydrocannabinol (HHC‐COOH), which appears as a mixture of the epimers 9*α*‐HHC‐COOH (**153**) and 9*β*‐HHC‐COOH (**154**). Due to its structural similarity to THC metabolite, commonly urinary screening panels show cross‐reactivity between THC and HHC metabolites, which can result in false positives.

**SCHEME 34:**
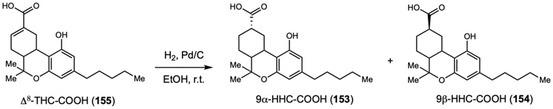
Synthesis of HHC‐COOH epimers via Pd/C‐catalyzed reduction of Δ^8^‐THC.

In 2024, Tsujikawa and coworkers [53] synthesized both HHC‐COOH epimers by Pd/C‐catalyzed reduction of 11‐nor‐9‐carboxy‐Δ^8^‐tetrahydrocannabinol (**155**) under hydrogen pressure (Scheme 34). The epimers were separated by preparative TLC and characterized by GC–MS and NMR spectroscopy. HHC‐COOH metabolites were tested in three commercially available immunoassays for THC detection and showed cross‐reactivity in all of them. Moreover, the *β*‐epimer exhibited higher reactivity than *α*, increasing the risk of false‐positive results in THC doping analyses.

## 4 Latent Fingerprint Detection and Visualization

The detection and visualization of crime scene latent fingerprints (LFPs), invisible patterns formed by chemical residues on an object's surface upon finger contact, are among the most important ways to identify suspects during an investigation. Three levels of fingerprint details are necessary for unequivocal individual recognition. The first level concerns the macroscopic overall pattern of a fingerprint, easily visible to the naked eye, which includes basic shapes classified as arches, loops, and whorls. The second level, or minutiae, refers to individual ridge characteristics such as terminations, bifurcations, and enclosures. The third level comprises microscopic elements within the ridge, including sweat pore locations, ridge width, ridge shape, and scars [8]. The techniques employed to visualize LFPs include the cyanoacrylate fuming method, fluorescent staining method, electrochemiluminescence imaging, Raman spectroscopy, mass spectrometry, and others. In forensic routine, the fluorescent staining method is widely adopted due to the low cost of stain reagents and the availability of portable equipment. In 2001, Tang and coworkers reported a fluorescent molecule, 1‐methyl‐1,2,3,4,5‐pentaphenylsilole, that exhibited strong emission in their aggregated state and was almost nonemissive in dilute solution [54]. This behavior, coined by them as “aggregation‐induced emission” (AIE), contrasts with the quenching by aggregation observed at concentrated solutions of traditional luminogens. In comparison to traditional planar luminogen structure, AIEgens typically possess twisted molecular conformations, which play a crucial role in their AIE photophysical properties and minimize *π*–*π* stacking interactions involved with aggregation‐caused quenching (ACQ) [55]. The pioneering application of tetraphenylethene for LFP visualization reported by Li and coworkers achieved second‐level information details in a few minutes under UV illumination, without a self‐quenching effect and with low background interference, since luminescence is generated by aggregates solely [56]. For LFP visualization, AIEgens employed should be non‐ or weakly emissive in solution at the working concentration and exhibit strong emission after binding to biochemical components left on the finger impression, usually lipids and fatty acids. Among the different mechanisms that explain this phenomenon, the restriction of intramolecular motion (RIM), a combination of restriction of intramolecular rotation (RIR) and restriction of intramolecular vibration (RIV) within the dye structure, is the most accepted [57]. In RIM, the aggregation blocks the nonradiative decay pathway, forcing the excited‐state energy to be released as radiative emission. Since the seminal work by Li and coworkers, several groups have turned their attention to improve resolution and safety of AIEgens. In 2020, Zhu and coworkers reported a water‐soluble probe based on triphenylamine with remarkable third‐level resolution, achieving several requirements of an ideal LFP developer [58]. Despite this successful example, more research has been conducted to design AIEgens with different luminescent properties, such as wavelength, lifetime, and quantum yield, to reduce or eliminate background interference. In this context, this section is organized into three main topics that join the selected examples from literature considering the scaffolds that confer AIE properties to each dye: triphenylamine derivatives, carbazole derivatives, and miscellaneous AIEgens scaffolds. Our objective is to present the synthetic routes used for these dyes and highlight their similarities. Further, we discussed the relation between the probe design and the functional improvements. Exceptionally, the scope of this section is limited to studies published in 2024 and 2025, due to the considerable number of recent contributions in this area, including multiple comprehensive reviews [8, 59, 60, 61].

### 4.1 Triphenylamine Derivatives

**SCHEME 35:**
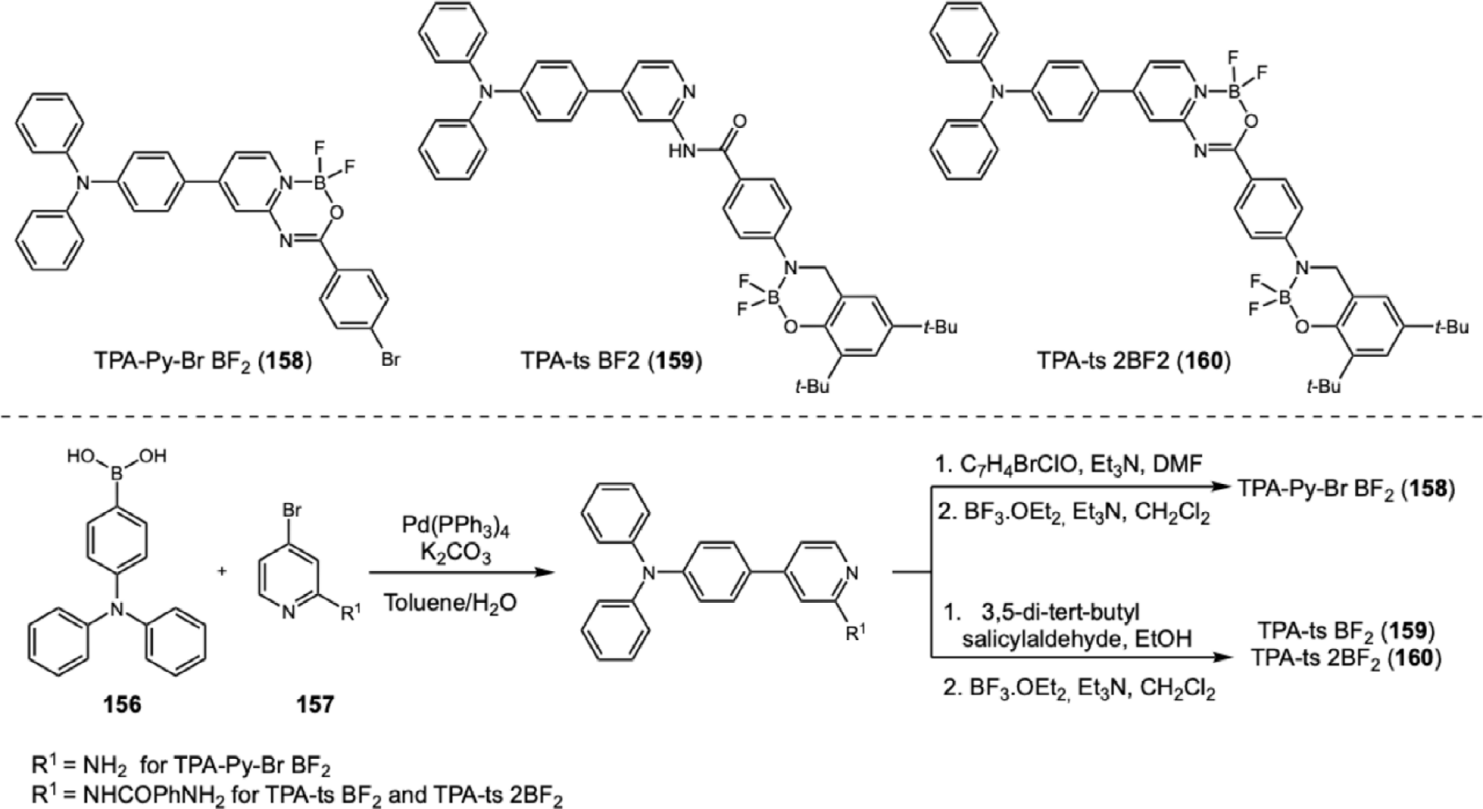
Suzuki coupling for the construction of TPA‐difluoroboron probes.

In 2024, Yang and coworkers developed a new TPA‐based probe displaying excellent AIEE activity and mechanofluorochromic (MFC) behavior (Scheme 35) [62]. The MFC behavior, switchable fluorescence under external mechanical stimulation, was one of the characteristics of the solid‐state luminescence and showed interesting applications for inkless writing and anticounterfeiting. The probe synthesis involves a Suzuki coupling of boronic acid‐TPA 156 to 4‐bromopyridin‐2‐amine (157). Next, the difluoroboron cycle was constructed after an amide bond installation on pyridine, followed by a treatment with boron trifluoride. Evaluation of LFP development was conducted by dripping a solution of 158 at 100 μM in water:tetrahydrofuran (2:1) onto a glass surface. The glass surface was washed with distilled water, left to dry in the shade, and photographed under a 365 nm UV lamp, revealing high‐resolution image with third‐level features (i.e., microscopic ridge details such as the size, shape, and location of sweat pores). Additionally, the solid powder was tested and showed potential for inkless writing.

The same research group reported another example demonstrating the contrasting behaviors of two TPA‐difluoroboron probes on water:tetrahydrofuran mixtures [63]. In this work, TPA‐ts‐BF2, a mono difluoroboron probe, and TPA‐ts‐2BF2, a bis‐difluoroboron probe, were synthesized in five steps. Firstly, the amidation between 2‐amino‐4‐bromopyridine (157) and 4‐nitrobenzoyl chloride was conducted, followed by reduction of nitro group, affording a key intermediate to the Suzuki coupling with boronic acid of TPA. Next, reaction with 3,5‐di‐tert‐butyl salicylaldehyde, formed an imine compound, which was treated with boron trifluoride to yield TPA‐ts‐BF2 (159) in 1.1% and TPA‐ts‐2BF2 (160) in 1.8%. While TPA‐ts‐2BF2 shows AIE with increasing water content in solution, TPA‐ts‐BF2 undergoes aggregation‐caused quenching (ACQ). Theoretical calculations indicate that the amide linkage makes the molecule adopt a conformation prone to aggregation. Evaluation of LFP development was conducted by spraying a solution of TPA‐ts‐2BF2 at 10 μM in water:tetrahydrofuran (2:1) on a glass surface. After 1 h, the glass was washed with distilled water and immersed in a beaker with water for 20 min, before being left to dry in the shade. Revelation under 365 nm UV lamp showed a high‐resolution image with third‐level features. MFC behavior was evaluated, revealing superior efficacy for TPA‐ts‐2BF2, suggesting that multiple boron fragments could improve solid‐state emission.

A remarkable advance was achieved by Liu, Zhu, and coworkers regarding the poor background contrast when LFPs are present on multicolored substrates [64]. In this work, four different colored BODIPY (161–164) dyes were designed and synthesized. BDP‐G (161) was synthesized via the classical BODIPY synthesis route, and three more BODIPY dyes were subsequently prepared through Knoevenagel reaction with aldehyde derivatives exhibiting different electron‐donating abilities (phenyl, methoxyphenyl, and triphenylamino groups) 162–164. As the electron‐donating ability increased, both the absorption and emission spectra exhibited progressive red shifts, resulting in a color gradient from green to yellow, orange, and red under 365 nm UV light irradiation. The evaluation of LFP development was performed with 10 μM solution of probes in water:ethanol (6:4) using spraying method for 10 s. The visualization under 365 nm showed high‐resolution definition with third‐level details on several substrates. Additionally, the evaluation on aluminum cans with various colored backgrounds showed that probe color modulation from green to red effectively addresses complex background colors, ensuring optimal detection with high contrast.

**SCHEME 36:**
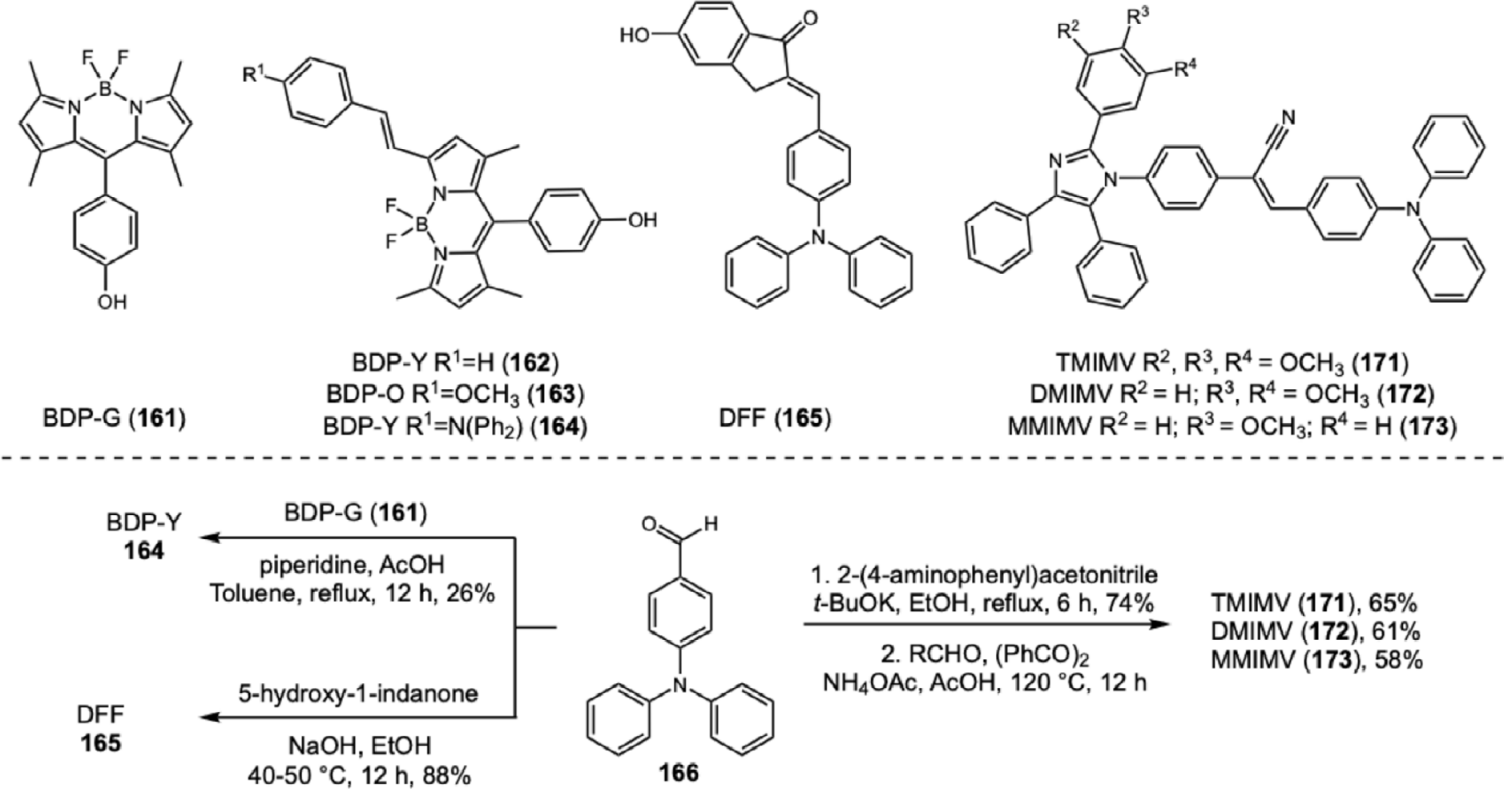
Aldol and Knoevenagel condensation on the synthesis of DFF, BODIPY, and TPA‐imidazole probes.

Hu and coworkers reported another example with a notable response to colored or fluorescent substrates. This developer consists of a TPA‐chalcone dye (**165**) combined with montmorillonite at 0.67% dye mass fraction (DFF‐MMT) [65]. The probe synthesis occurs in two steps. First, the triphenylamine was submitted to a Vilsmeier–Haack reaction, originating the corresponding aromatic aldehyde **166**. Then, chalcone is obtained through an aldol condensation between **166** and 5‐hydroxy‐1‐indanone (Scheme 36) in 88% yield. Evaluation of DFF‐MMT for developing LPFs across a wide range of substrates demonstrates your exceptional potential for real‐time development with high‐resolution differentiation between level 2 and level 3 information. Upon contact with LFPs, the dye interacts with sebaceous residues, changing its original orange color to yellow. This unique characteristic surpasses background interferences on challenging substrates, such as colored and fluorescent substrates or leather materials. Moreover, aged LFPs, with 15 days and 30 days, were developed exhibiting minimal changes under 365 nm UV light and can be stored for 21 months, preserving their main characteristics. Biological assays showed that DFF has low cytotoxicity and does not damage DNA.

Further, Mao, Zhang, and coworkers reported an interesting study with three TPA‐based probes (**167–169**) containing different groups to investigate the effect of lipophilicity on LFP imaging performance [66]. These probes were synthesized in 2 steps. First, 4‐bromo‐1,8‐naphthalic anhydride undergoes a nucleophilic substitution reaction with amine compounds. The resulting products were submitted to a Suzuki coupling with 4‐(diphenylamino)phenyl boronic acid (**156**), originating TPA‐NA‐Pr (**167**), TPA‐NA‐OH (**168**), and TPA‐NA‐COOH (**169**). Evaluation of AIE properties revealed inactivation of TPA‐NA‐COOH by quenching with the increase of water amount in the solution mixture. Among the chemical components of fingerprint, only glyceryl trioleate and oleic acid interact with TPA‐NA‐Pr and TPA‐NA‐OH. Evaluation of LFP imaging was conducted with a 20 μM TPA‐NA‐Pr solution in water:acetonitrile (1:1) and a 30 μM TPA‐NA‐OH solution in water:ethanol (1:1). High‐quality images of LFPs, on different materials, were produced by immersion or spraying method after 2–5 s under 405 nm LED irradiation. These probe solutions stored for 2 months could be successfully used for LFP imaging with high‐resolution details. Moreover, LFP imaging with TPA‐NA‐Pr and TPA‐NA‐OH was kept visible for a period up to 60 days.

**SCHEME 37:**
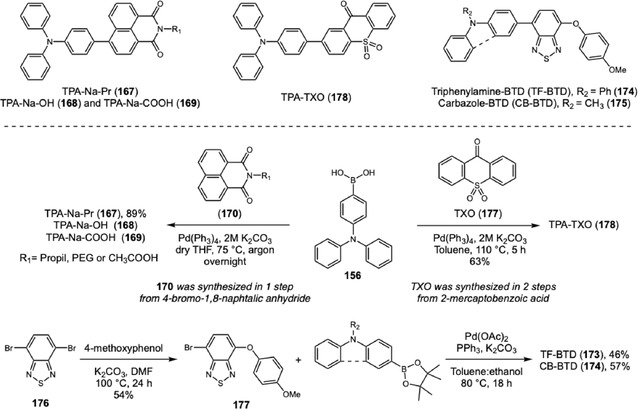
Suzuki coupling on the synthesis of TPA‐naphtalimide, TPA‐TXO, and benzothidiazole probes.

Iyer and coworkers developed three fluorescent probes based on the combination of TPA, stilbene, and imidazole [67]. The synthesis starts with a Knoevenagel condensation reaction, followed by a multicomponent reaction to construct three stilbene‐coupled imidazole probes (**171–173**) with distinct methoxy group configurations: TMIMV (**171**), DMIMV (**172**), and MMIMV (**173**) (Scheme 37). Fluorescence enhancement through AIE is greater for MMIMV compared with DMIMV and TMIMV, owing to the presence of more methoxy groups, which alter hydrophilicity and interfere with AIE properties. The powder dusting method was employed for fingerprint analysis using MMIMV, DMIMV, and TMIMV powders on various substrates. The mild imaging process, broad applicability, and high‐contrast images with minimal background fluorescence demonstrate the potential for forensic application. Finally, these probes have also been implemented for anticounterfeiting applications.

Considering the ability of TPA to improve the interactions with the lipid fraction on LFPs, Limberger and coworkers designed a probe with TPA in combination with aryloxy‐benzothidazole (TF‐BTD) (**174**) and a structurally related probe with carbazole instead of TPA (CB‐BTD) (**175**) [68]. For the synthesis, a dibromo‐BTD **176** was submitted to a substitution reaction with 4‐methoxyphenol, leading to the monobrominated intermediate **177** in 54% yield. Subsequently, this intermediate was conjugated to TPA or methylcarbazole by a Suzuki coupling, affording CB‐BTD and TF‐BTD in 57% and 46% yields, respectively. The ability of both CB‐BTD and TF‐BTD to develop LFPs on nonporous substrates was evaluated. After dipping the LFPs in a 700 μM solution of CB‐BTD in water:acetone (9:1) or in a 700 μM solution of TF‐BTD in water:acetone (7:1), high‐resolution images were produced with first and second‐level details for CB‐BTD and with third‐level details for TF‐BTD.

Concurrently, Zhuang and coworkers reported the development of a donor–acceptor probe composed of TPA and 9‐H‐thioxanthen‐9‐one‐10,10‐dioxide (TXO) [69]. As mentioned above, the key reaction for TPA‐based probe construction is typically a Suzuki coupling with TPA‐boronic acid. According to the literature, TXO was synthesized in two steps, yielding TPA‐TXO (178) in 34%. For LFP imaging, different substrates were immersed in a 100 μM solution of TPA‐TXO in water:ethanol and visualized under 420 nm irradiation; the best results were achieved with 37.5% and 50% of water. The probe tends to precipitate into the latter solution; on the other hand, it remains stable with 37.5% of water solution and could be successfully applied after 20 days of storage. In this work, the authors also demonstrate broad substrate compatibility and robust performance under relatively extreme conditions, including the visualization of aged LFPs.

**SCHEME 38:**
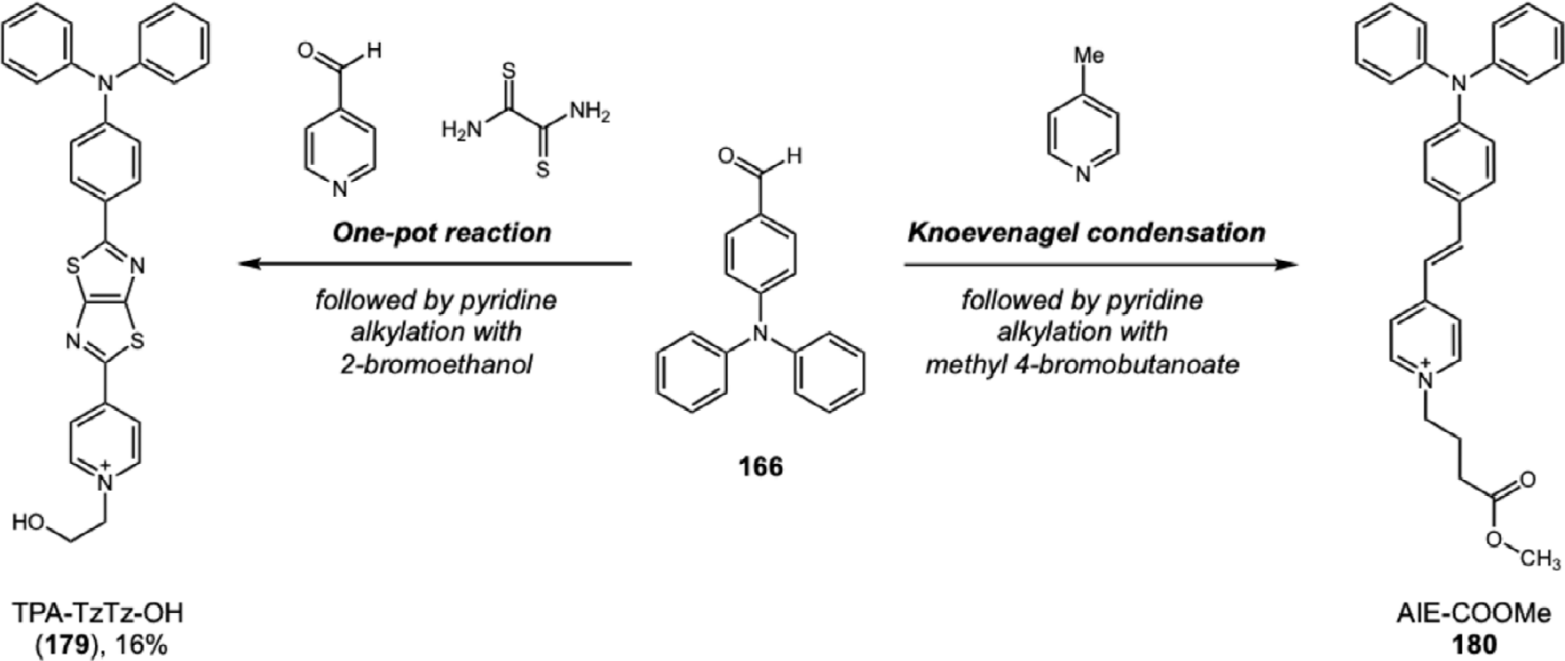
Synthesis of AIE‐COOMe via Knoevenagel condensation and one‐pot reaction for TPA‐TzTz‐OH construction.

In 2025, Yuan, Gao, Zheng, and coworkers achieved a breakthrough in LFP development research by developing TPA‐thiazolo[5,4‐d]thiazole (TPA‐TzTz‐OH) (179) (Scheme 38). This water‐soluble probe enables visualization of LFPs in pure water upon visible‐light excitation, providing a greener and safer alternative [70]. TPA‐TzTz‐OH was synthesized via a one‐pot condensation reaction, followed by N‐alkylation with 2‐bromoethanol, yielding the product in 16%. Investigation of probe fluorescence against different substances commonly found in LFPs exhibited an intense fluorescence exclusively in the presence of oleic acid and cholesterol. Further, synthesis of TPA‐TzTz‐OH analogs revealed the importance of the hydroxyl group for cholesterol recognition and the role of the pyridinium cation in its interaction with oleic acid, driven by electrostatic attraction. Additionally, TzTz has a substantial contribution on molecule RIM and acts as a *π*‐bridge, strengthening intramolecular charge transfer (ICT) from triphenylamine to the pyridinium, which reflects on AIE properties. Evaluation of LFP development on multisubstrates by immersion in an aqueous solution of TPA‐TzTz‐OH at 100 μM for 60 s, followed by visualization under 425 nm irradiation, produced images with third‐level resolution. Alternatively, LFP visualization can be performed by spraying an aqueous solution of TPA‐TzTz‐OH at 100 μM for 30 s, then exposing to 425 nm light. Interestingly, prolonged spraying until 60 s resulted in diminished resolution, likely due to excess reagent accumulation. Moreover, this probe exhibits low cytotoxicity, retains efficacy for aged fingerprints, and could be successfully applied after storage in the dark for 1 month.

Most AIE materials bind exclusively to the lipidic residues on LFPs, due to their hydrophobic nature. Unfortunately, the LFP visualization process often uses organic solvents, which cause lipid dissolution and partial loss, leading to faint LFP images. In addition, the chemical components of LFPs break down, becoming dynamic and unpredictable for different individuals after deposition on substrates. To address the diminished resolution resulting from these drawbacks, Li and coworkers designed an AIE probe with a multitarget response (Scheme 38) [71]. The synthesis consists of a Knoevenagel condensation of TPA‐benzaldehyde with methylpyridine, followed by alkylation of pyridine with methyl 4‐bromobutanoate to yield the AIE‐COOMe probe (**180**). The development of LFPs was evaluated on different substrates, including tinfoil, steel, glass, etc., by immersion in a 30 μM aqueous solution of AIE‐COOMe for 60 s. All the images produced showed high contrast and reached third‐level resolution details after visualization under 405‐nm irradiation. Compared with other TPA‐based probes, AIE‐COOMe extends beyond the usual interactions with lipids and anchorage to negatively charged fatty acids, also showing affinity for amino acids and proteins. Surprisingly, similar probes containing a carboxyl or a hydroxyl group instead of a methyl ester do not present this affinity. DFT calculations supported the idea that the methyl group could increase the electron density of the adjacent carbonyl group, making it easier to form hydrogen bonds than its carboxylic acid analog. The probe efficacy was also confirmed in the development of aged LFPs and after storage for 365 days. However, the probe solution at 20 μM exhibited significant toxicity.

### 4.2 Carbazole Derivatives

**SCHEME 39:**
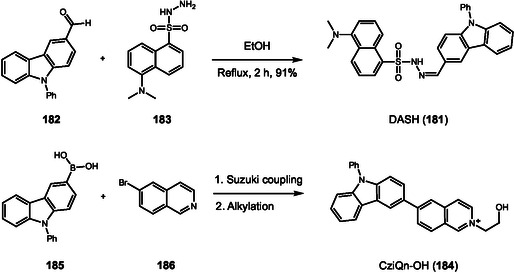
DASH synthesis by Schiff‐base formation and CziQn‐OH synthesis by Suzuki coupling.

In 2025, Dhir, Jaiswal, and coworkers designed and synthesized a carbazole‐dansyl conjugate, DASH (181), which presents application in date rape drug detection and latent fingerprint development (Scheme 39) [72]. The Schiff‐base compound, DASH, was synthesized in 91% yield by the reaction of 9‐phenyl‐9H‐carbazole‐3‐carboxaldehyde 182 with dansyl hydrazine (183). LFP development was evaluated on porous and non‐porous substrates by dusting the solid, composed by 10% weight of DASH on nanosílica, and removing the excess gently with a fine brush. The LFP was subsequently examined under a 365 nm UV lamp, revealing third‐level resolution. In drug‐facilitated sexual assaults, beverages are commonly adulterated with drugs like gamma‐butyrolactone (GBL) and gamma‐valerolactone (GVL), due to their colorless and odorless nature. To evaluate the efficacy of detecting these drugs, approximately 7 mg of DASH were placed in a flask with GBL (1 μL of drug sample) or GVL (1.28 μL of drug sample). The observation under 365 nm irradiation showed a change of luminescence from blue to yellowish green in both cases. The same result was obtained when the experiment was performed for both drugs spiked in a beverage, demonstrating the potential of this probe.

Further, Di, Zhang, Zhao, Yang, and coworkers present a process for the acquisition of fingerprint evidence using an amphiphilic dye, CziQn‐OH (**184**), and a commercially available compression nebulizer (Scheme 39) [73]. The dye synthesis involves a Suzuki coupling of (9‐phenyl‐9H‐carbazol‐3‐yl)boronic acid (**185**) with 6‐bromoisoquinoline (**186**). The obtained isoquinoline carbazole derivative was then alkylated with 2‐bromoethanol to produce CziQn‐OH. Evaluation of LFP development across several substrates, including a stainless‐steel blade to simulate a real crime scene, by nebulizing the dye at 100 μM in aqueous solution for 6 s, produced high‐definition images with third‐level features under 365 nm. LFP staining by soaking with an aqueous solution of CziQn‐OH is virtually instantaneous, taking only 0.8 s. Compared to previously reported probes, it exhibits a high lipid‐water partition coefficient, which promotes a rapid migration from the aqueous to the lipid phase. The DFT calculation reveals a distinct energy discrepancy for CziQn‐OH in water and 1‐octanol, which is significantly higher than those of reported amphiphilic AIE developers, such as TPA‐1OH, CPAB, LFP‐red, and LFP‐yellow. From the synthesis and evaluation of three analogs with similar structures, the authors confirm the role of a positive charge on nitrogen for the solubility in water, of carbazole for AIE activity, and of hydroxyl plus carbazole for high‐definition LFP imaging due to interactions with lipidic fraction. Experimental analysis showed that CziQn‐OH has a multitargeted action owing to interactions with cholesterol, lactic acid, oleic acid, and triacetin via van der Waals forces. Moreover, the probe efficacy was also confirmed for the development of aged LFPs, for the use after storage for 3 months, and for DNA collection and amplification analysis.

### 4.3 Miscellaneous AIEgens Scaffolds

**SCHEME 40:**
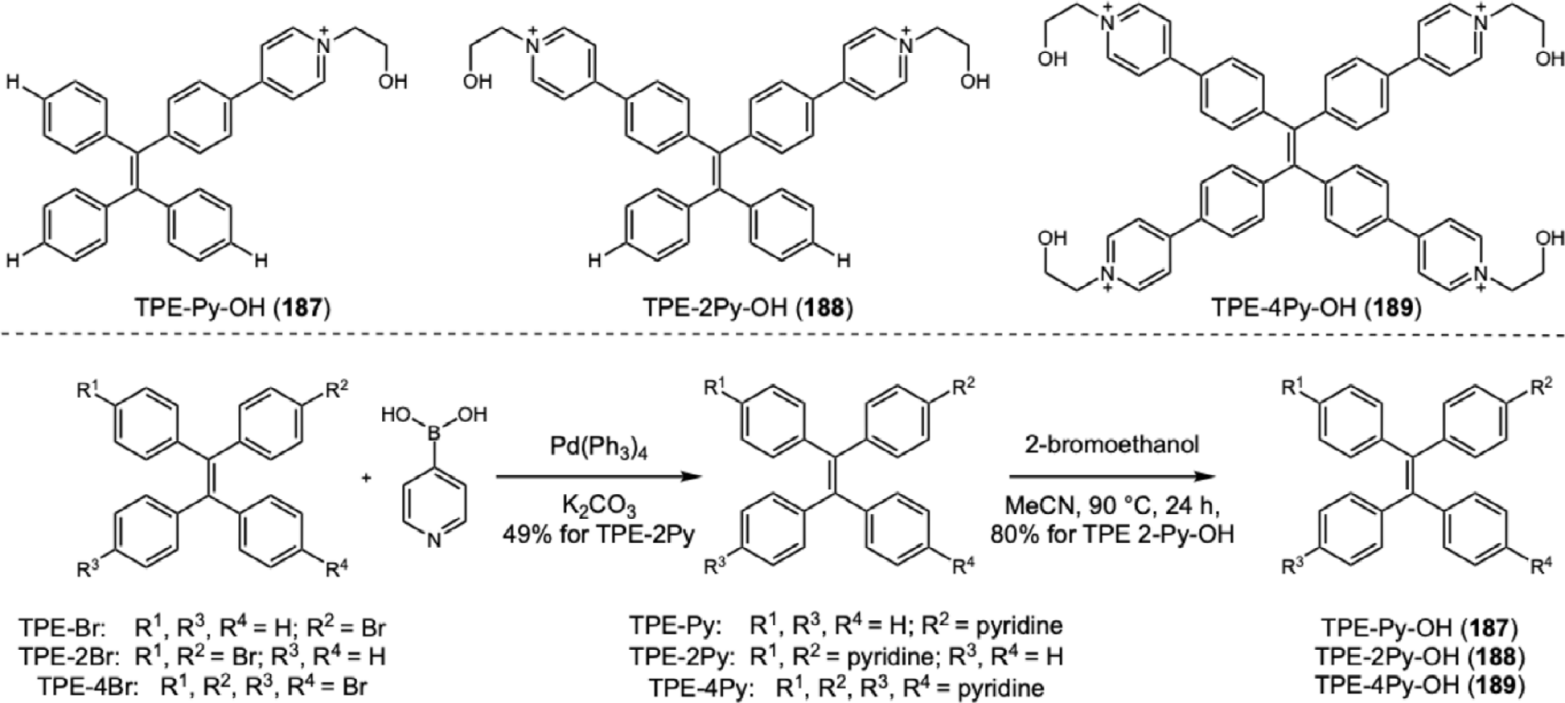
Tetraphenylethene probe synthesis by Suzuki coupling.

In 2024, Wang and coworkers designed a series of water‐soluble probes based on tetraphenylethene (TPE) in combination with pyridinium salts (Scheme 40) [74]. In this way, the authors are pursuing a TPE probe that presents high contrast LFP imaging due to selective fluorescence emission only after binding to fingerprint residues. Additionally, the use of water solution prevents the dissolution of LFP lipidic fraction. The synthesis involves a Suzuki coupling to combine TPE core to pyridine, followed by an alkylation with 2‐bromoethanol to afford TPE‐Py‐OH (187), TPE‐2Py‐2OH (188), and TPE‐4Py‐4OH (189). For evaluation of LFP development, only TPE‐2Py‐2OH was tested due to its very weak fluorescence emission on water. LFPs on several substrates were immersed in aqueous solution of TPE‐2Py‐2OH at 30 μM for 10 s and visualized under 365‐nm irradiation, reaching high resolution definition. Moreover, the probe was demonstrated to be effective for aged LFPs imaging, can be stored for 3 months preserving their activity, does not present cytotoxicity at 30 μM concentration, and can be also applied on antibiotics detection in aqueous solution.

**SCHEME 41:**
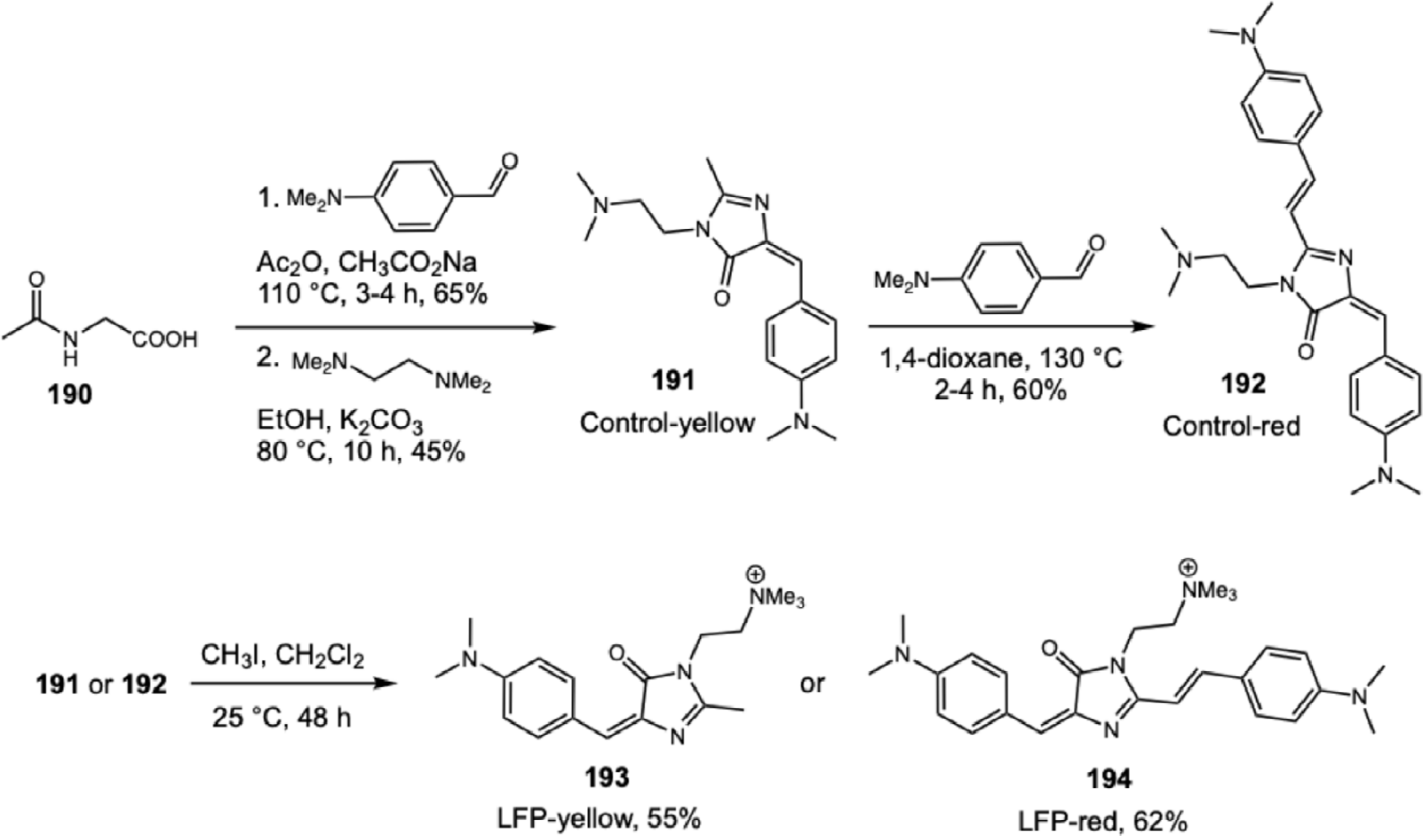
Green fluorescent protein‐based probes through classical Erlenmeyer azlactone synthesis.

James, Huang, Wu, and coworkers developed four Green Fluorescent Protein‐based probes [75]. These probes were synthesized in 3 or 4 steps. Initially, a classical Erlenmeyer azlactone synthesis from acetylglycine (**190**) and *p*‐dimethylaminobenzaldehyde resulted in azlactone in 65% yield. Further, the azlactone is converted to imidazolinone control yellow **191** in 45% yield under conditions shown in Scheme 41. The condensation reaction of control yellow **191** with *p*‐dimethylaminobenzaldehyde furnished control red imidazolinone **192** in 60% yield. Finally, alkylation of aliphatic amine gives LFP‐yellow (**193**) and LFP‐red (**194**) in 55% and 62% yield, respectively. The efficacy of LFP visualization on typical substrates, including ceramics, glass, and complex surfaces of objects, such as the adhesive side of tapes, rough surfaces of semi‐absorbent stones, and wood, was evaluated using a portable ultrasonic atomizer, designed by the authors, to spray the dyes in aqueous solutions for 10 s. After this period, LFPs were visualized under 445‐nm irradiation and photographed with a system designed by the authors, generating clear and high‐contrast images. In terms of detail resolution, LFP‐Red produced better images than LFP‐Yellow across all substrates, and both probes achieved level 3 minutia discrimination for selected LFPs on plastic and steel. The efficacy of LFP‐Red is likely due to its long *π*‐conjugated system, which reduces background signal from the substrates. Additionally, the longer LFP Red chain makes it much more flexible, enabling LFP‐Red to make multiple contacts with components in the LFPs, thereby improving resolution. Additionally, the lack of pyridinium groups or metal ions on these dyes prevents DNA contamination. STR analysis confirmed that no interference was observed when these dyes were used for DNA extraction and identification.

**SCHEME 42:**
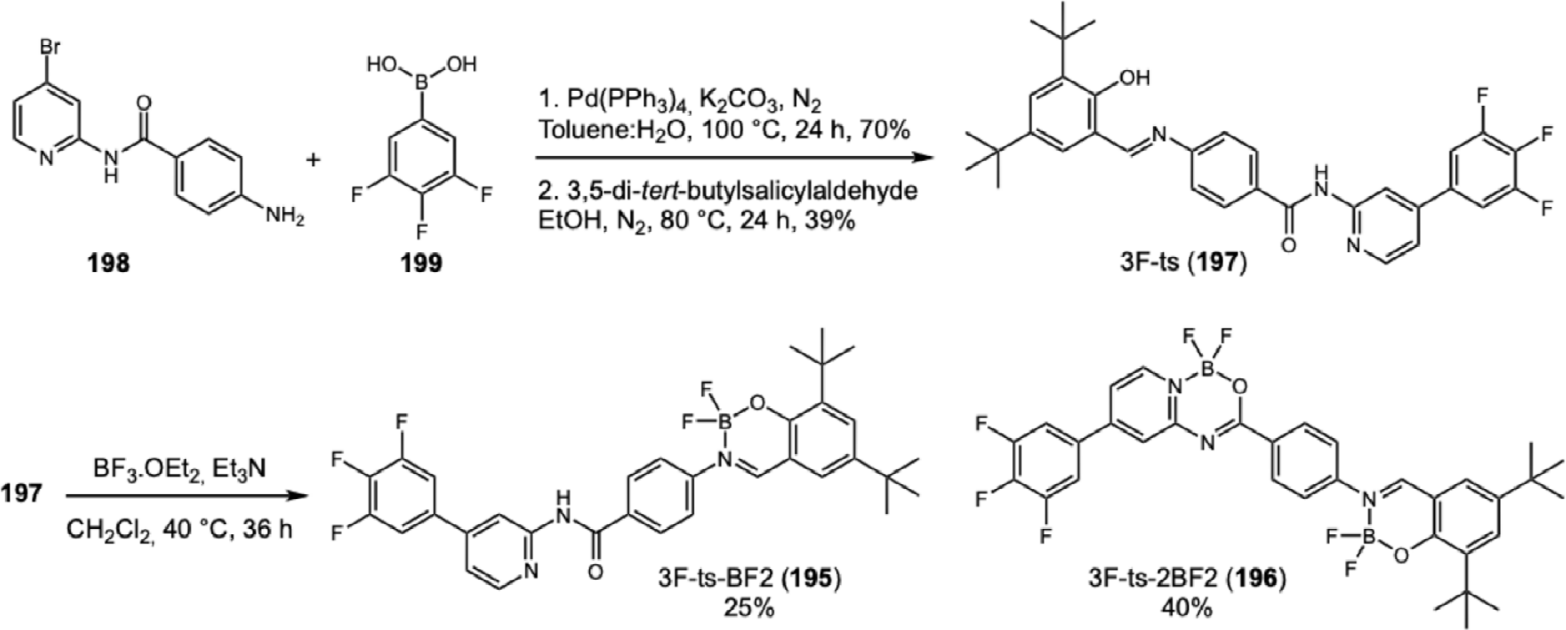
Suzuki coupling followed for Schiff‐base formation on the construction of difluoroboron probes.

As aforementioned above, Yang and coworkers explored difluoroboron compounds in combination with TPA for probes that exhibit MFC behavior and high‐resolution LFP visualization (Scheme 42). Here, the authors designed and synthesized two difluoroboron compounds 3F‐ts‐BF2 (**195**) and 3F‐ts‐2BF2 (**196**) in combination with 3,4,5‐trifluorophenyl (**197**) in 3 steps [76]. First, 4‐amino‐N‐(4‐bromopyridin‐2‐yl)benzamide (**198**) was submitted to a Suzuki coupling with trifluoroaryl boronic acid (**199**). Next, reaction with 3,5‐di‐*tert*‐butyl salicylaldehyde formed an imine intermediate, which was treated with boron trifluoride to yield 3F‐ts‐BF2 in 6.8% and 3F‐ts‐2BF2 in 10.9%. Evaluation of efficacy of LFP development was conducted by spraying a solution of 100 μM of **195** and **196** in water:acetonitrile 8:2 on a glass surface containing the LFP. After 30 min, the glass was washed with distilled water and left to dry in the shade. Revelation under 365 nm UV lamp showed a high‐resolution image with third‐level features for both probes. On more challenging materials, the utilization of 3F‐ts‐BF2 provides better image resolution than 3F‐ts‐2BF2. The authors speculated that uncoordinated amide moiety in 3F‐ts‐2BF2 structure could be responsible for stronger luminescence, owing to its electron‐rich nature. On the other hand, the amide could easily be hydrated with water and oily components of fingerprint secretion, improving the adhesion of 3F‐ts‐BF2. Besides their potential for forensic investigation, all difluoroboron probes reported in this minireview use hazardous solvents for inkless writing or require laborious postprocessing for LFP development, complicating their use at the crime scene. Moreover, there is no data about their cytotoxicity or DNA damage.

## 5 Summary and Outlook

In recent years, organic synthesis has contributed significantly to the advancement of forensic science, enhancing analytical reliability, supporting criminal investigations, and enabling rapid responses to the continuous emergence of new psychoactive substances. In the field of drug identification, synthetic approaches have made it possible to accurately differentiate isomers, reconstruct clandestine synthetic routes, and produce analytical standards. These materials, essential for ensuring traceability, comparability, and precision in forensic analyses, cover a wide range of pharmacological classes, including hallucinogens, stimulants, antidepressants, opioids, and anesthetics.

In forensic toxicology, the synthesis of metabolite standards has become increasingly crucial for confirming substance exposure and elucidating metabolic pathways, particularly in a scenario characterized by the rapid proliferation of new psychoactive compounds. The ability to isolate and confirm metabolites of synthetic opioids, amphetamine‐derived stimulants, and artificial cannabinoids strengthens the capacity for analytical confirmation, reduces the occurrence of false positives, and improves toxicological interpretation in clinical, criminal, and human‐performance settings.

In the field of latent fingerprint detection, the area with the greatest number of developments in recent years, the creation of fluorescent probes based on aggregation‐induced emission materials has transformed the technological landscape of fingerprint visualization. Molecules containing triphenylamine, carbazole, difluoroboron dipyrromethene, tetraphenylethylene, and chromophores inspired by fluorescent proteins demonstrate how rational molecular design can overcome long‐standing limitations, such as substrate interference, loss of ultrafine details, and incompatibility with subsequent genetic analysis.

Despite these advances, organic synthesis remains less explored in the forensic context than it could be and progresses more slowly than the broader field of modern synthetic chemistry. Contemporary tools, such as direct C—H bond activation, late‐stage functionalization, molecular editing, continuous‐flow synthesis, and automated platforms, are still rarely incorporated into the analytical and investigative needs of forensic science. This gap represents a strategic opportunity to deepen the integration of synthetic innovation with emerging forensic challenges, particularly considering the rapid evolution of the illicit synthetic drug market.

Looking ahead, closer collaboration among organic chemists, toxicologists, forensic analysts, and regulatory agencies will be essential to translate laboratory innovations into robust, routine protocols. There is substantial space for progress in new synthetic methodologies and processes, the construction of impurity libraries, the integration of machine‐learning tools, and the development of fully aqueous, sustainable probes suitable for on‐site use. In this scenario, organic synthesis is no longer merely a means of constructing molecules but becomes a strategic instrument for investigation, prevention, and the strengthening of public safety.

## Funding

This study was supported by Coordenação de Aperfeiçoamento de Pessoal de Nível Superior (88887.516472/2020‐00).

## Conflicts of Interest

The authors declare no conflicts of interest.
